# Conversion of rainforest into oil palm and rubber plantations affects the functional composition of litter and soil Collembola

**DOI:** 10.1002/ece3.7881

**Published:** 2021-07-13

**Authors:** Winda Ika Susanti, Tamara Bartels, Valentyna Krashevska, Rahayu Widyastuti, Louis Deharveng, Stefan Scheu, Anton Potapov

**Affiliations:** ^1^ J.F. Blumenbach Institute of Zoology and Anthropology University of Göttingen Goettingen Germany; ^2^ Department of Soil Sciences and Land Resources Institut Pertanian Bogor (IPB) Bogor Indonesia; ^3^ UMR7205 CNRS Muséum national d'Histoire naturelle Institut de Systématique, Évolution, Biodiversité (ISYEB) Sorbonne Université Paris France; ^4^ Centre of Biodiversity and Sustainable Land Use Göttingen Germany; ^5^ Russian Academy of Sciences A.N. Severtsov Institute of Ecology and Evolution Moscow Russia

**Keywords:** agricultural plantation, community structure, rainforest conversion, springtail, trait composition

## Abstract

Rainforest conversion and expansion of plantations in tropical regions are associated with changes in animal communities and biodiversity decline. In soil, Collembola are one of the most numerous invertebrate groups that affect the functioning of microbial communities and support arthropod predators. Despite that, information on the impact of changes in land use in the tropics on species and trait composition of Collembola communities is very limited. We investigated the response of Collembola to the conversion of rainforest into rubber agroforestry (“jungle rubber”), rubber, and oil palm plantations in Jambi Province (Sumatra, Indonesia), a region which experienced one of the strongest recent deforestation globally. Collembola were sampled in 2013 and 2016 from the litter and soil layer using heat extraction, and environmental factors were measured (litter C/N ratio, pH, water content, composition of microbial community and predator abundance). In the litter layer, density and species richness in plantation systems were 25%–38% and 30%–40% lower, respectively, than in rainforest. However, in the soil layer, density, species richness, and trait diversity of Collembola were only slightly affected by land‐use change, contrasting the response of many other animal groups. Species and trait composition of Collembola communities in litter and soil differed between each of the land‐use systems. Water content and pH were identified as main factors related to the differences in species and trait composition in both litter and soil, followed by the density of micro‐ and macropredators. Dominant species of Collembola in rainforest and jungle rubber were characterized by small body size, absence of furca, and absence of intense pigmentation, while in plantations, larger species with long furca and diffuse or patterned pigmentation were more abundant. Overall, land‐use change negatively affected Collembola communities in the litter layer, but its impact was lower in the soil layer. Several pantropical genera of Collembola (i.e., *Isotomiella*, *Pseudosinella*, and *Folsomides*) dominated across land‐use systems, reflecting their high environmental adaptability and/or efficient dispersal, calling for studies on their ecology and genetic diversity. The decline in species richness and density of litter‐dwelling Collembola with the conversion of rainforest into plantation systems calls for management practices mitigating negative effects of the deterioration of the litter layer in rubber plantations, but even more in oil palm plantations.

## Graphical abstract

Conversion of rainforests into plantation systems altered the composition of Collembola communities with potential consequences for decomposition processes and other ecosystem services they provide. The community composition of Collembola differed to a similar extent between rainforest, jungle rubber, and monoculture plantations suggesting that Collembola communities are sensitive to even moderate changes in land use.

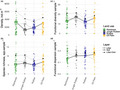

## 1 INTRODUCTION

Agricultural intensification in Indonesia is associated with deforestation, which has increased greatly in the last 30 years and is predicted to continue (Gatto et al., 2015; Koh & Ghazoul, 2010). The conversion of tropical rainforest into plantations is associated with the degradation and destruction of habitats, which is among the most significant and immediate threats to biodiversity worldwide (Titeux et al., 2016). Environmental changes and habitat degradation eliminate or alter ecological niches resulting in a loss of biodiversity and associated changes in ecosystem functioning (Barnes et al., 2014; Clough et al., 2016; Fitzherbert et al., 2008; Gilbert, 2012). Experiencing massive deforestation in the last decades, Sumatra represents a model region for investigating the effect of rainforest conversion into plantation systems on biodiversity and ecosystem functioning at local and regional scales (Clough et al., 2016; Drescher et al., 2016).

Lowland rainforest in Jambi Province, Sumatra, has been converted in large into oil palm (16% of total area) and rubber plantations (12%) (Gatto et al., 2015). Conversion of rainforest into oil palm and rubber plantations is associated with a strong decline in plant and animal diversity above the ground (Clough et al., 2016; Fitzherbert et al., 2008; Rembold et al., 2017), but also alters soil habitats and detrimentally affects soil‐associated biodiversity, as shown for nematodes (Krashevska et al., 2019), testate amoebae (Krashevska et al., 2016), ground spiders (Potapov et al., 2020), and litter macroinvertebrates (Barnes et al., 2014). Extensively managed agricultural systems, such as rubber agroforests, have potential compared to intensively managed systems, such as monoculture plantations of rubber and oil palm, to mitigate detrimental effects of the conversion of rainforest into agricultural production systems on biodiversity and community composition (Krashevska et al., 2016; Nazarreta et al., 2020; Schulz et al., 2019). However, information on effects of conversion of rainforest into monoculture plantations and agroforestry systems is lacking for many groups of soil fauna, including Collembola.

Collembola is a dominant group of soil animals globally, being very abundant in forest soils (Devi et al., 2011; Hopkin, 1997; Rusek, 1998). Collembola significantly affect soil microbial communities, nutrient cycling, and soil fertility by feeding on soil microorganisms and dead organic matter (Coulibaly et al., 2019; Potapov et al., 2020; Rusek, 1998). In particular, the presence of different ecological groups of Collembola (soil or surface dwellers) can affect microbial‐driven ecosystem processes, such as decomposition and nutrient cycling (Coulibaly et al., 2019). In temperate regions, agricultural intensification (Sousa et al., 2006) and forest plantations (Deharveng, 1996) typically are associated with a decrease in species richness and density, as well as a strong alteration in the community composition of Collembola as compared to natural forests. The few studies existing from tropical regions suggest that effects of land use on Collembola communities are negative, as shown, for example, for monoculture coffee plantations in Mexico (Rojas et al., 2009) and rubber and peach plantations in Amazonia (Martius et al., 2004). However, very few studies investigated the impact of land‐use change on Collembola in other tropical regions, including Southeast Asia. A study from Sumatra indicated that the conversion of rainforest into oil palm and rubber plantations negatively affects Collembola density and genus‐level diversity and composition (Widrializa et al., 2015), but consequences for the functional diversity and species composition remained unexplored.

Since the biology of tropical soil invertebrates is poorly studied, their functional roles and responses might be inferred from approximations, with functional traits (properties of species that govern their effects on or response to their environment) being a promising approach (Pey et al., 2014; Violle et al., 2007). Trait‐based approaches have been suggested to be more advantageous than species‐based approaches since they may provide more mechanistic and generalizable links between organisms and their environment (Gagic et al., 2015). Collembola species display a wide variation in traits that can provide an insightful tool for assessing their response to land‐use changes (Van Straalen et al., 2008). Traits usually considered for Collembola are morphological characters assumed to be connected to adaptations to environmental conditions. They often are combined into composite traits such as “life forms,” linked to particular microhabitats, that is, size of furca, number of ocelli, length of antennae, pigmentation, and presence of scales (Salmon & Ponge, 2012; Vandewalle et al., 2010). As shown in temperate ecosystems, Collembola species and communities show trait‐related responses to a variety of environmental factors, like changes in soil chemistry, microhabitat configuration, vegetation cover, and agricultural practices, but primarily Collembola traits reflect the soil water content and pH (Chagnon et al., 2001; Chauvat, Ponge, et al., 2007; Chauvat, Wolters, et al., 2007; De Boer et al., 2012; Son et al., 2009). However, it remains unknown which factors drive changes in species and trait composition of Collembola communities in tropical rainforests and plantation systems.

In this study, we investigated the species and trait composition of Collembola communities from litter and soil in rainforest, rubber agroforestry (“jungle rubber”), rubber, and oil palm plantations, in Jambi Province, Sumatra, Indonesia. Based on previous studies on the impact of land‐use changes on invertebrate communities in this region (Barnes et al., 2014; Krashevska et al., 2016, 2019; Potapov et al., 2020), we hypothesized that (a) rainforest and jungle rubber have higher total density, species richness, and functional diversity of Collembola than monoculture plantations, (b) community composition of Collembola changes with land use, with the changes being most pronounced between more natural ecosystems (rainforest and jungle rubber) and more intensively used ecosystems (oil palm and rubber plantations), (c) similar to temperate ecosystems, pH and water content are the most important factors correlated with the composition of Collembola in rainforest and plantations systems, and (d) functional trait composition of Collembola communities varies more predictably with environmental factors than species composition because of environment‐specific trait responses favoring certain functional groups of species.

## 2 MATERIALS AND METHODS

### 2.1 Site description

The study was conducted in the framework of the EFForTS project investigating in a comprehensive way ecological and socioeconomic changes associated with the transformation of rainforest into plantation systems (http://www.uni‐goettingen.de/en/310995.html). Soil and litter samples were taken in lowland rainforest, jungle rubber, rubber (*Hevea brasiliensis*) plantations, and oil palm (*Elaeis guineensis*) plantations, located in Jambi Province, southwest Sumatra, Indonesia. The climate is tropical and humid with moderate variations between the rainy season from October to April and the dry season from June to September; between 1991 and 2011, mean annual temperature was 26.7 ± 0.2°C and mean annual precipitation 2,235 ± 381 mm (Drescher et al., 2016). The study sites were located at similar altitude varying from 50 to 100 m a. s. l. in two landscapes, Harapan and Bukit Duabelas region; the overall area of the sampling sites was about 80 km in diameter with the distance between sites in the two regions being <40 km (for more details, see Drescher et al., 2016). Rainforest was included to represent baseline conditions allowing to evaluate changes due to the conversion into agricultural plantations. Except in jungle rubber, samples from upland and riparian sites (being at least partly flooded during the rainy season) were studied. Soils in the Harapan region mainly comprise loamy Acrisols of low fertility, whereas in the Bukit Duabelas region, the major soil type is clay Acrisol (Allen et al., 2015; Kotowska et al., 2015). Two regions and upland and riparian sites were included in the analysis to increase the variation in environmental factors considered (water regimes, soil types) and thereby the generality of our results.

Rubber and oil palm plantations were intensively managed monocultures of 6–16 and 8–15 years, respectively (Drescher et al., 2016). Typically, oil palm plantations were established after clearing and burning of jungle rubber, whereas rubber plantations were established after logging of rainforest (Allen et al., 2015). Rubber and oil palm plantations were fertilized once in the rainy season and once in the dry season with NPK complete fertilizer (i.e., Phonska and Mahkota), potassium chloride (KCl), and urea (CO(NH_2_)_2_). Additionally, manual and chemical weeding took place throughout the year in both rubber and oil palm plantations. The most commonly used herbicides were Gramoxone and Roundup, applied at an average rate of 2–5 L ha year^−1^ (Allen et al., 2015; Clough et al., 2016; Kotowska et al., 2015). More details on management practices of the studied smallholder monoculture plantations are described in Allen et al. (2015).

### 2.2 Sampling procedure

**FIGURE 1:**
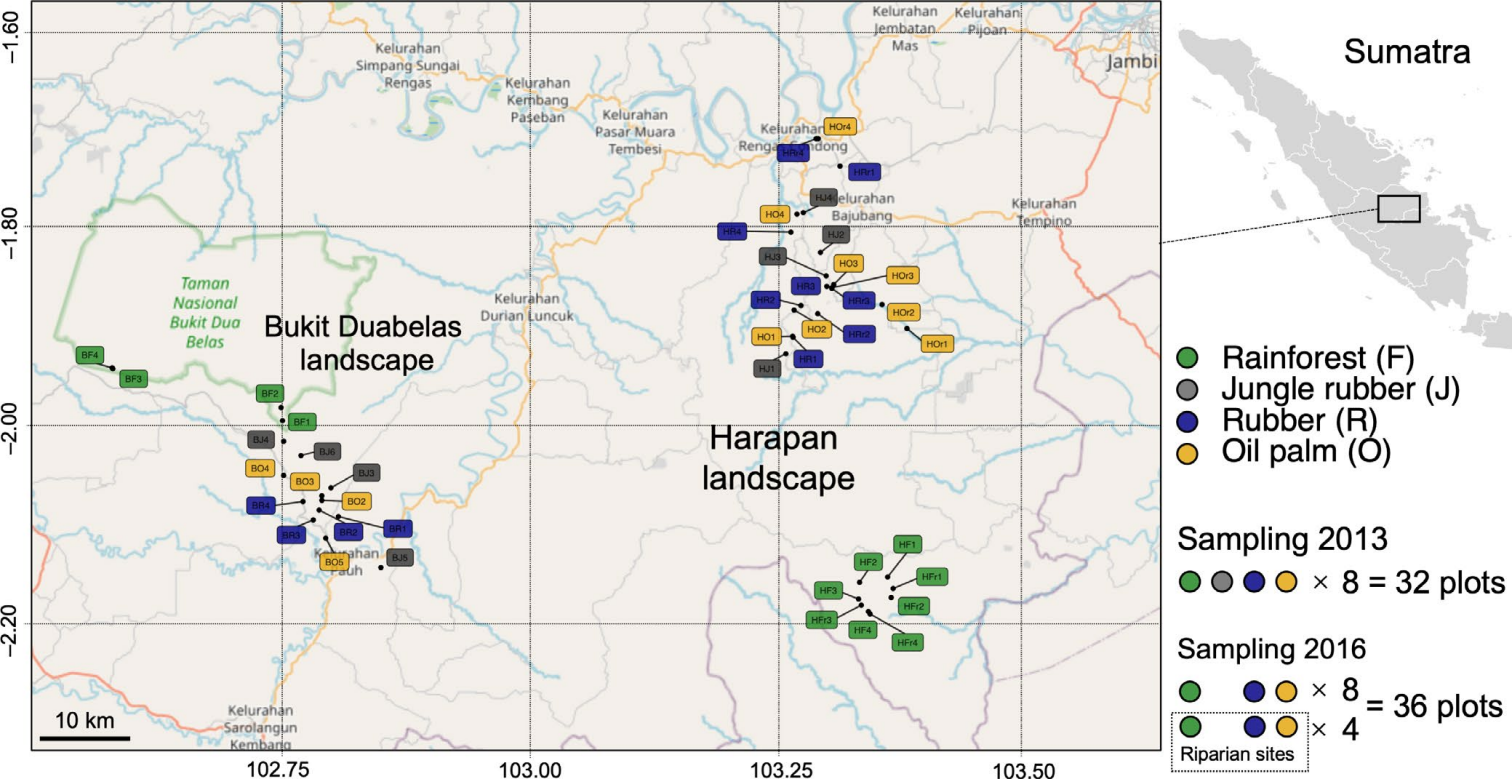
Map of the sampling locations in two landscapes (Bukit Duabelas and Harapan) in 2013 and 2016; for details, see text

Samples were taken in October 2013 and October 2016 within 50 × 50 m² plots established at each study site (see Drescher et al., 2016) with a distance of ca. 500 meters minimum, but usually more than 1 kilometer, between the plots. Two different years were used as temporal replicates increasing the variability of environmental factors considered and therefore the generality of our results. In 2013, samples were taken in natural rainforest, jungle rubber, rubber, and oil palm plantations in two regions with four replicates each, resulting in 32 plots in total (2 regions × 4 land‐use systems × 4 replicates). In 2016, samples were taken in rainforest, rubber, and oil palm plantations in two regions (2 regions × 3 land‐use systems × 4 replicates) with additional rainforest, rubber, and oil palm plantations in riparian sites of the Harapan region with four replicates each (1 region × 3 land‐use systems × 4 replicates), resulting in 36 plots in total (Figure 1).

From each plot at each sampling event, one randomly positioned soil core was taken. Each core measured 16 × 16 cm taken to a depth of 5 cm of the mineral soil. Litter and soil layer were separated in the field and extracted independently. Samples were transported to the laboratory for extraction of soil animals. Animals were extracted by heat for 5–10 days until the substrate was completely dry (Kempson et al., 1963), collected in dimethyleneglycol–water solution (1:1), and stored in 70% ethanol until further processing. Environmental variables were measured in composite samples of each litter and soil (five cores per plot within a radius of ca. 2 m around the soil animal sample), including abiotic factors (pH, water content, C‐to‐N ratio) and biotic factors (microbial community composition in litter and soil as indicated by phospholipid fatty acids; Krashevska et al., 2015; V. Krashevska, unpublished data; see Table A1). Litter and soil pH (CaCl_2_) were measured using a digital pH meter. Aliquots of litter and soil material were dried at 65℃ for 72 hr, milled, and analyzed for total C and N concentrations using an elemental analyzer (Carlo Erba). Water content of litter and soil was determined gravimetrically (for more details on environmental factors, see Krashevska et al., 2015).

The extracted animals were sorted to high‐rank taxonomic groups (mostly orders; Potapov et al., 2019). From these data, we used the abundance of potential predators (Mesostigmata, Araneae, and Formicidae) and potential competitors (Oribatida) of Collembola as biotic factors potentially affecting their abundance and community composition; for details, see Table A1. We sorted and identified all individuals of Collembola from the 2013 samples (in total 4,069 individuals), and we took a random subsample of 25% of individuals from the 2016 samples (in total 986 individuals). This unequal sampling effort is expected to affect species richness (accounted in the model), but not density and proportional community composition estimates.

### 2.3 Species identification

Collembola were sorted into morphogroups under a dissecting microscope (Zeiss, Stemi 508) at 50× magnification, based on basic morphological characters (body size, body shape, morphology of furca, antennae and number of eyes). A number of individuals of each morphogroup from each sample were subsequently cleared with Nesbitt solution on a heating plate (50°C) for 3–10 min. Then, the animals were mounted on slides with Hoyer's solution (for details, see Glime & Wagner, 2017). Collembola were identified to species level using a compound microscope (Axiovert 35, Zeiss) at maximum 400× magnification, using the checklist for Indonesian Collembola (Suhardjono et al., 2012) and additional articles containing keys for Collembola of southeast Asia, particularly Indonesia (Mateos & Greenslade, 2015; Potapov, 2012; Potapov & Starostenko, 2002). Due to the poorly described fauna, in many cases we had to assign individuals to morphospecies without Latin binomials (in total 70% of all identified species). Below, we refer to all identifications as “species” for simplicity. Whenever possible, juvenile specimens were ascribed to species by comparing with adults or subadults. The full list of traits and species is given in Table S1. Along with the identification, each species was described using a set of morphological traits (Table 1). The same selected set of traits was used for each species across land‐use systems for statistical analysis, including presence/absence of empodial appendage, sucking/chewing mouthpart, presence/absence of postantennal organ (PAO), presence/absence of scales, elongated/normal abdomen IV, elongated/spherical abdomen, separate/fused abdominal segments, presence/absence of furca, straight/curved and short/long furca, presence/absence of pigmentation, diffuse/patterned and intensive/light pigmentation, normal/modified antennae, and small/medium/large body size (Table 1). Data on Collembola species, including their traits and pictures, were uploaded to the open virtual research environment “Ecotaxonomy” (http://ecotaxonomy.org).

**TABLE 1:** Collembola traits used in the study and their potential functions

Trait	Potential function	Trait states	References
Abdominal modifications	Excretion, digestion, reproduction, colonization of specific (micro)habitats	Abdomen IV elongated, spherical abdomen, fused abdomen	Hopkin (1997), Suhardjono et al. (2012)
Antennae modifications	Sensory function, modified antennae in some genera (clasping antennae) also used for mating	Clasping antennae, subdivided antennae I and II, subdivided antennae III and IV, antennae I very long, antennae IV shorter than III	Hopkin (1997), Suhardjono et al. (2012)
Body size	Metabolic demands, dispersal ability, predator–prey interactions	Total length from the front of the head to the end of the abdomen: small: <0.7 mm, medium: 0.7–1.2 mm, large: >1.2 mm	Hopkin (1997)
Empodial appendage	Helping to walk, particularly on wet surfaces	Present, absent	Christiansen (1965)
Furca development	Active dispersal abilities of species and predator avoidance	Furca absent, short, straight furca, long furca	Hopkin (1997), Ponge and Salmon (2013)
Mouthparts	Type of food or feeding strategy. Small size of the apical mouth opening and no molar plate indicate absence of capability to convey solid food particles	Molar plate present or absent (piercing‐sucking mouthparts)	Hopkin (1997), Suhardjono et al. (2012), Adams (1979)
Postantennal organ (PAO)	Sensory function, particularly in olfaction	PAO absent, PAO simple, PAO complex	Hopkin (1997), Suhardjono et al. (2012)
Pigmentation	UV protection, thermodynamic buffering and signaling, camouflage	Absent, diffuse, intensive, patterned	Hopkin (1997), Salmon et al. (2014)
Scales	Desiccation protection, thermodynamic buffering and signaling, potentially predation avoidance	Present, absent	Hopkin (1997), Salmon et al. (2014), Hawes and Greenslade (2015)

Note

For more details on the traits, see http://ecotaxonomy.org/traits. Combinations of different traits (life form) are related to trophic niches of species and thus their role in ecosystems (Potapov et al., 2016).

### 2.4 Statistical analysis

In all analyses, we used individual soil samples as replicates (litter and soil separately), that is, *n* = 64 in 2013 (32 plots × 2 layers, litter and soil) and *n* = 72 in 2016 (36 plots × 2 layers, litter and soil). Area‐based density and species richness were analyzed as count data (individuals/species per sample). In addition, we calculated density per gram of carbon in litter and soil using the data from Krashevska et al. (2015). Statistical analyses were conducted using R version 3.5.3 (R Core Team, 2017) unless stated otherwise.

To test whether rainforest and jungle rubber have higher total density, species richness, and functional diversity of Collembola than rubber and oil palm plantations (hypothesis 1), we inspected the effect of land‐use system (rainforest, jungle rubber, rubber, oil palm), layer (litter, soil), land‐use system–layer interaction, hydrological position (riparian, upland), years (2013 and 2016), and landscapes (Harapan and Bukit) on total density, species richness, functional diversity (FD), and functional dispersion (FDis) of Collembola. Generalized linear models with these factors were run using *glmer.nb* (negative binomial distribution and optimizer = "bobyqa" option) in the *lme4* package v. 1.1‐21 (Bates et al., 2015). Soil core was included as random effect to account for interdependency of cosampled soil and litter layers (Zuur et al., 2009). We run several models of increasing complexity and the final model was selected using *ANOVA* comparison of more complex versus simplified models based on AIC values. Significance was evaluated using the Wald chi‐square test with *ANOVA* in the *car* package v. 3.0‐2 (Fox & Weisberg, 2019). Pairwise differences between treatments were assessed using Tukey contrasts by applying *glth* and *cld* in the *multcomp* package v. 1.4‐10 (Hothorn et al., 2008). The model selection procedure is described in more detail in Tables A1, A2, A3, A4, A5, A6.

To calculate FD, traits were coded as binary zero and one variables except for body length which was coded as numerical variable scaled between 0 and 1. The trait matrix was transformed into a dendrogram using *hclust* and then used to calculate FD in each community (i.e., sample) using *treedive* in the *vegan* package v. 2.5‐5 (Oksanen et al., 2019) (Figure S1). FDis was calculated using *fdisp* in the *FD* package and represented the weighted average distance of all species to the weighted community centroid in multidimensional space (Laliberté & Legendre, 2010). FDis is another measure of FD that, in contrast to FD, accounts for species abundances. Since the distributions of FD and FDis data were close to normal, we used *lmer* instead of *glmer.nb*. The final models were selected using the same procedure as described above.

To test whether community composition of Collembola changes with land use (hypothesis 2), we applied linear discriminant analyses (LDA) to assess differences in community composition of Collembola species and traits among land‐use systems. All species and trait values were weighted based on their relative abundance in the community. For that, we used the number of individuals of certain species or with a certain trait state. To reduce the effect of random species occurrence and unequal sampling efforts between 2013 and 2016, only species occurring in at least three plots were included in the analysis (Tables S2 and S3). Nonmetric multidimensional scaling (*metaMDS*) with six dimensions was done before continuing with LDA. LDA was done with the *MASS* package using all six axes from the NMDS (Venables & Ripley, 2002). Wilks' lambda and *p*‐values determined the effect of land‐use system on community composition and were calculated using *MANOVA* in the *pander* package v. 0.6.3 (Daróczi & Tsegelskyi, 2018). After this, pairwise tests between land‐use systems were conducted using *HotellingsT2* in the *ICSNP* package v. 1.1‐1 (Nordhausen et al., 2018). To numerically estimate distances among communities from different land‐use systems, squared Mahalanobis distances (MD^2^) between land‐use systems were calculated using the *mahal* function in the *HDMD* package v. 1.2 (McFerrin, 2013).

To inspect effects of environmental factors on species and traits (hypotheses 3 and 4), we used multivariate community analyses (canonical correspondence analysis (CCA) and redundancy analysis (RDA)). We applied forward selection CCA as implemented in CANOCO 5.02 (ter Braak & Smilauer, 2012) to explore the correlation between species composition of communities and environmental variables across the two sampling years. CCAs were performed because the lengths of gradients were 4.0 *SD* units for litter and 3.4 *SD* units for soil indicating unimodal species–environment relationships (Jan Leaps, 2003). Only species occurring in at least two plots in each land use were included in the analyses (Tables S2 and S3). The following environmental variables were included in the CCA that were a priori assumed to affect Collembola community composition: C‐to‐N ratio, pH(CaCl_2_) value, water content (%), sum of phospholipid fatty acid (PLFA) marker lipids of Gram‐positive bacteria (i15:0, a15:0, i16:0, i17:0) and Gram‐negative bacteria (2OH 12:0, 2OH 14:0, 16:1ω7, cy17:0, 2OH 16:0, cy19:0, 2OH 10:0), fungal PLFA marker (18:2ω6,9), relative PLFA marker of algae (20:5ω3), relative marker of arbuscular mycorrhizal fungi based on the neutral lipid fatty acid (NLFA) 16:1ω5c, abundance of potential predators (Mesostigmata, Araneae, and Formicidae), and abundance of potential competitors (Oribatida) (Table A1). Monte Carlo tests (999 permutations) were performed to evaluate the overall model significance and the significance of environmental variables and individual axes. Since the global test with all environmental variables was significant, we used forward selection to identify the most important environmental variables affecting Collembola communities. The forward selection procedure was stopped if a variable reached a level of significance >0.05. We additionally tested for spatial autocorrelation in community data by relating *pcnm* spatial vectors (*vegan* package in R) to species matrix using CCA (*cca* function in R). To do this test, we separately analyzed data from 2013 and 2016 after pooling soil and litter data from the same soil cores. Although some spatial patterns were observed visually, the results of *ANOVA* showed no significant spatial effects (*p* = 0.143, adjusted *R* ^2^ = 0.03 in 2013 and *p* = 0.141, adjusted *R* ^2^ = 0.03 in 2016).

Correlations between trait composition of communities (Tables S4 and S5) and environmental variables were analyzed using RDA as implemented in CANOCO 5.02 (ter Braak & Smilauer, 2012). RDA instead of CCA was performed because gradients of the trait data were 2.5 *SD* units for litter and 2.0 *SD* units for soil indicating linear trait–environment relationships (Jan Leaps, 2003). Hellinger standardization, recommended for RDA, was applied to the data prior to the analysis. The same twelve environmental variables as in CCA (see above) were included in the RDA, and the same procedures were used to assess significance of the environmental factors included. Land‐use systems, regions, and years were included as silent variables not affecting the ordination and displayed on both CCA and RDA figures.

## 3 RESULTS

### 3.1 Density, species richness, functional diversity (FD), and functional dispersion (FDis)

**FIGURE 2:**
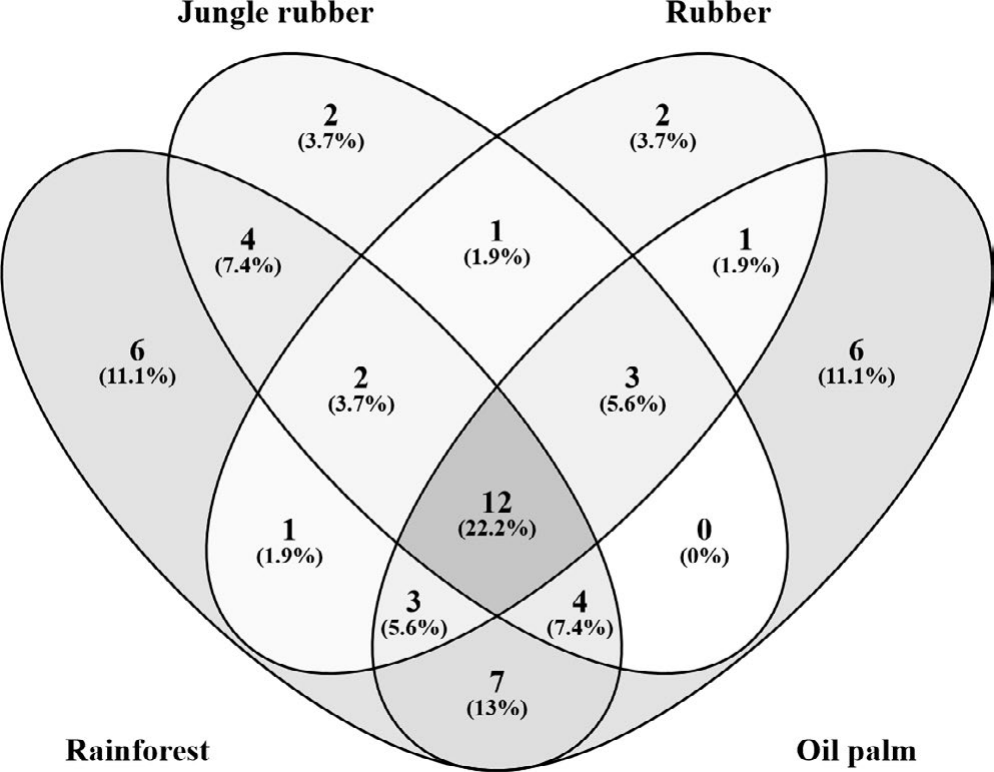
Venn diagram of species in different land‐use systems (rainforest, jungle rubber, rubber, and oil palm plantations)

**FIGURE 3:**
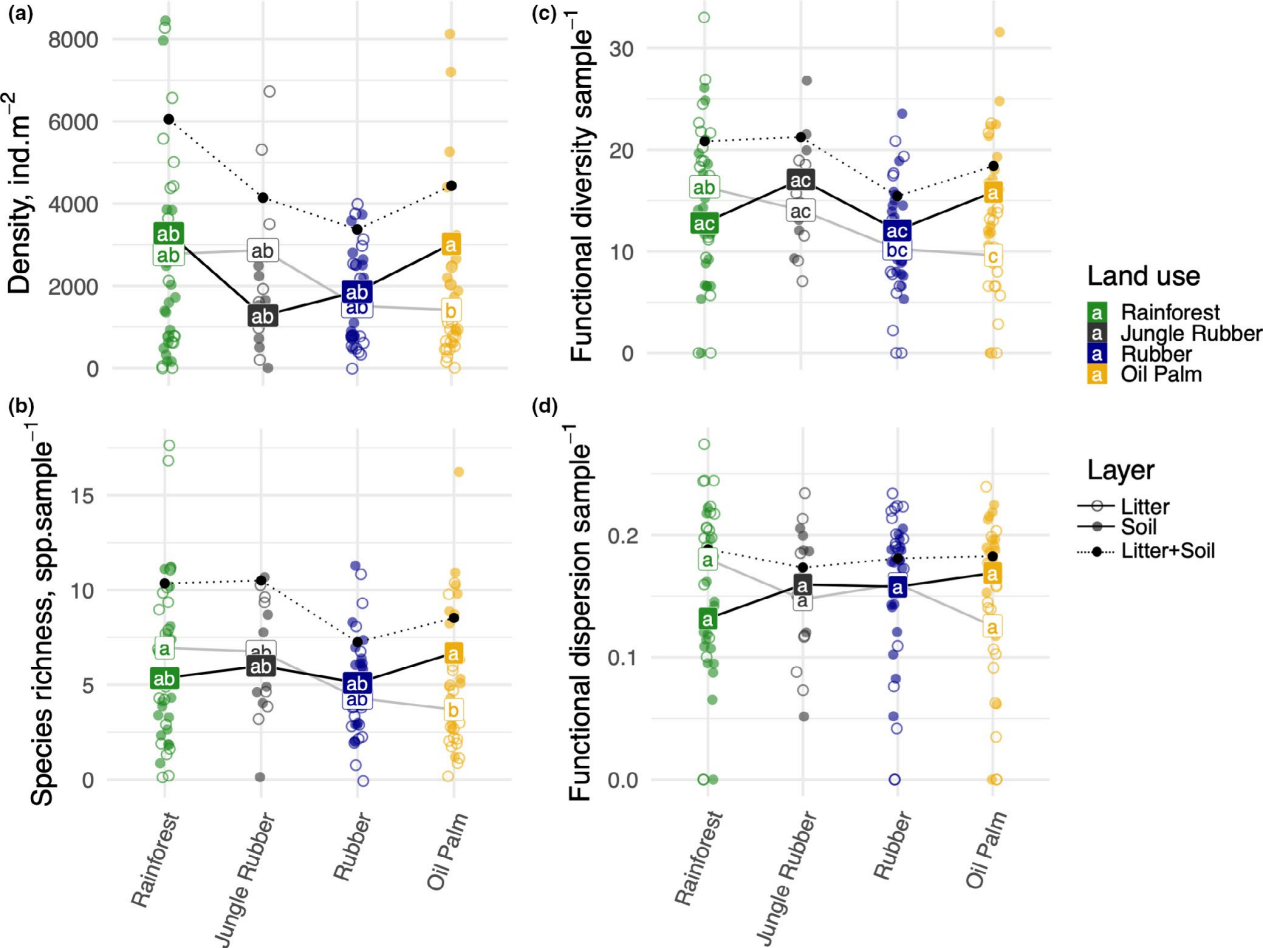
Density and species richness of Collembola in different land‐use systems across sampling years. (a) Density of Collembola per square meter, (b) number of Collembola species per sample (256 cm^2^), (c) functional diversity (FD) of Collembola communities per sample, and (d) functional dispersion (FDis) of Collembola communities per sample. Each soil core was divided into litter and soil layers (0–5 cm), and these layers were treated as replicates and presented as separate points in the figure (open points—litter, filled points—soil). Labels connected by solid lines show mean values for litter and soil separately (white labels—litter, black labels—soil). Mean values across layers and systems sharing the same letter are not significantly different for the given variable (Tukey contrasts). Black points connected with dotted lines show mean values for litter and soil combined (sum of density, newly calculated after combining layers for species richness, FD and FDis)

In total, 5,055 individuals were assigned to 54 species from 27 genera and 13 families across all land‐use systems and two sampling years with 22.2% of total species found across all land‐use systems, 11.1% only in rainforest, 3.7% only in jungle rubber, 3.7% only in rubber plantations, and 11.1% only in oil palm plantations, while the remaining ~48% were found in two or three land‐use systems (Figure 2). Rarefaction curves of the samples indicated that the number of species saturated in jungle rubber and rubber plantations in 2013 and in all systems in 2016 was close to saturation in rainforest and oil palm plantations in 2013 (Figure S1). Number of Collembola per unit area was 66% higher in litter than in soil, but was not significantly different among the land‐use systems (Figure 3a; Table 2). Across the two sampling years, differences in density and species richness among land‐use systems were moderate, and the main effect was the land‐use system × layer interaction due to significantly lower density (−50%) and species richness (−30%) of Collembola in litter than in soil of oil palm plantations than in rainforest (Figure 3b,c; Table 2). Although post hoc pairwise comparisons did not show significant differences, both density and species richness of Collembola in the litter layer tended to be higher in jungle rubber and rainforest than in rubber and oil palm plantations (−25 to −40%) (Figure 3b,c). Density and species richness of Collembola in the soil layer did not differ significantly among land‐use systems, being even slightly higher in oil palm plantations than in the other land‐use systems.

**TABLE 2:** Wald chi‐square test on the effect of layer, land‐use system, year, riparian, landscape, and their interaction on characteristics of Collembola communities based on mixed‐effects models

Factor	D.f.	Density (ind./m^2^)	Species richness (per sample)	Functional diversity (FD per sample)	Functional dispersion (FDis per sample)
Layer	1	2.7	1.4	2.9	0.1
Land‐use system	3	6.7	7.5	6.4	1.2
Year	1	*–*	48.7***	29.4***	3.8
Riparian	1	*–*	*–*	0.1	*–*
Landscape	1	3.5	*–*	3.6	*–*
Layer ⨯ Land‐use system	3	11.8*	14.5**	14.0**	12.7**

Note

Chi‐square values are shown; *p < 0.05; **p < 0.01; ***p < 0.001; and “–” factor not selected by the model.

Factors that were not selected by the model based on AIC are denoted by dashes.

Numbers in bold indicate significant values.

FD in the litter layer was higher (23%–26%) in litter of rainforest than in litter of plantations. By contrast, FD in the soil layer did not differ significantly among the land‐use systems (Figure 3d; Table 2). Both species richness and FD were strongly affected by year due to different sampling effort in the 2 years (see Methods). FDis did not differ significantly among land‐use systems according to post hoc comparisons, but it was generally highest in the litter layer in rainforest and lowest in the litter layer in oil palm plantations (significant land‐use system × layer interaction; Table 2). In addition, number of Collembola per gram of carbon was higher in litter than in soil, but was not significantly different among the land‐use systems (Table A5).

### 3.2 Species and trait composition of communities

**FIGURE 4:**
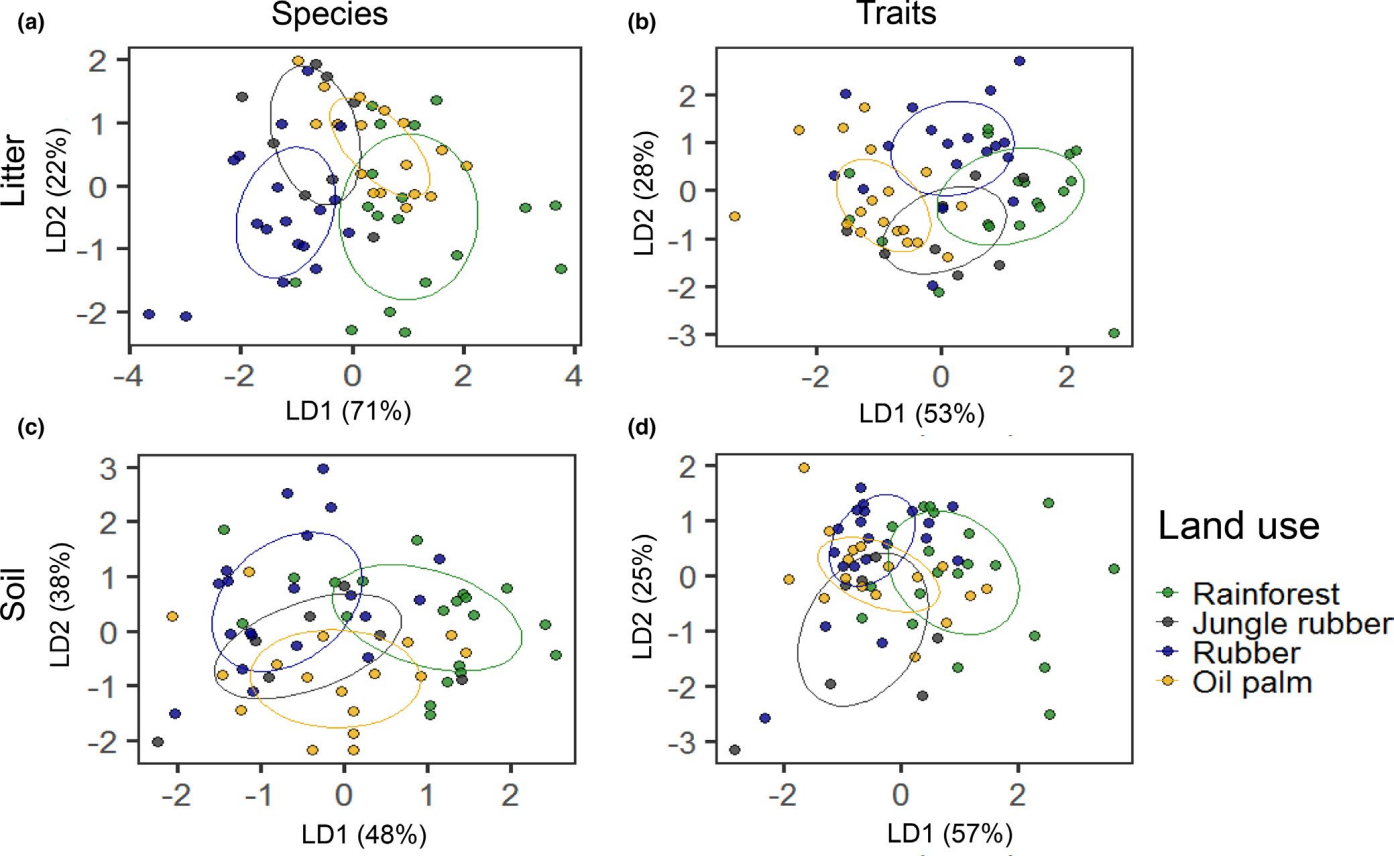
Linear discriminant analysis (LDA) of species (a, c) and trait composition (b, d) of Collembola communities in litter (a, b) and soil (c, d); data pooled for sampling years (2013 and 2016). Land‐use system was used as grouping variable. Ellipses were calculated using the *MASS* package in *R* to visualize the grouping of species or traits in the different land‐use systems. Each point represents a sample

**FIGURE A2:**
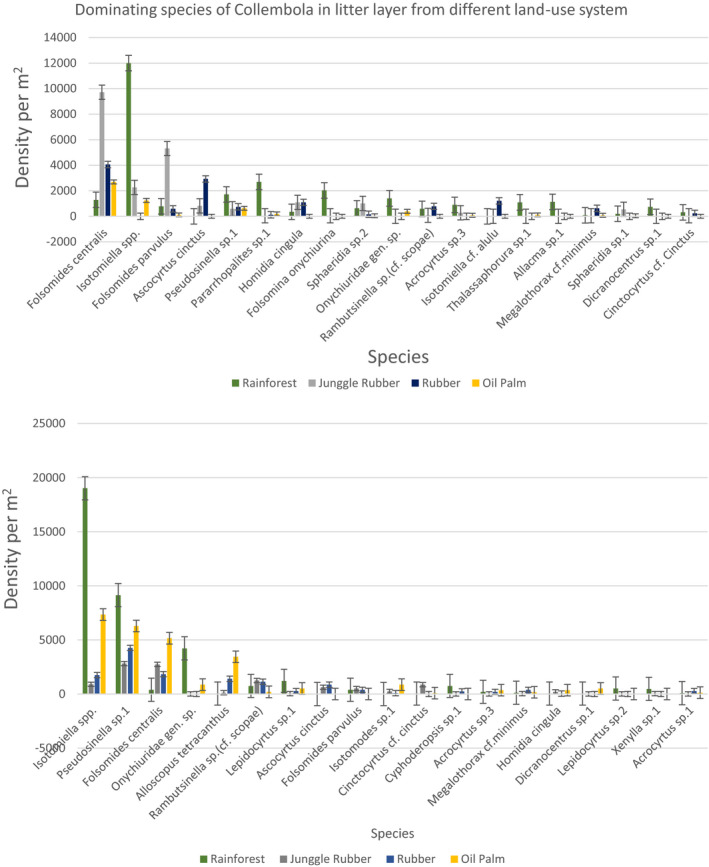
Dominating species of Collembola in litter and soil layer from different land‐use systems

**FIGURE A3:**
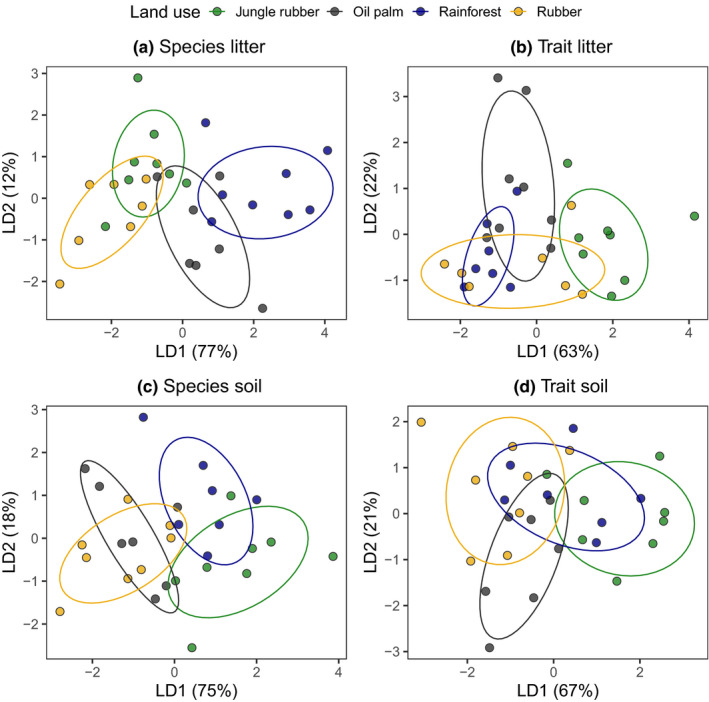
Linear discriminant analysis of species (a, c) and trait composition (b, d) of Collembola communities in the litter (a, b) and soil layer (c, d) in 2013. Land‐use system was used as grouping variable. Each point represents a sample

**FIGURE A4:**
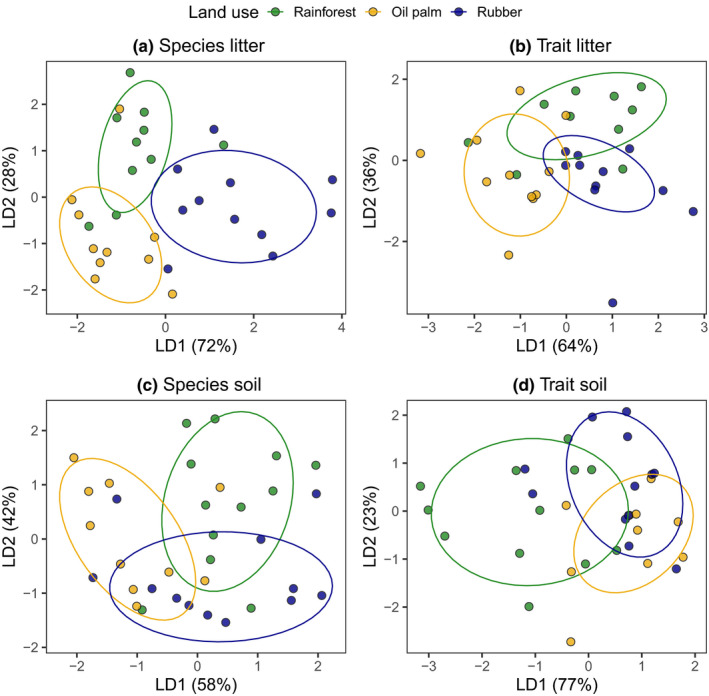
Linear discriminant analysis of species (a, c) and trait composition (b, d) of Collembola communities in the litter (a, b) and soil layer (c, d) in 2016. Land‐use system was used as grouping variable. Each point represents a sample

Of the 54 species identified, *Folsomides centralis*, *Isotomiella* spp., and *Pseudosinella* sp.1 were most abundant across land‐use systems (Figure A2). Land use had a pronounced effect on the species composition of Collembola communities in the litter layer, with the first LDA axis explaining 71% of the variation (Wilks' lambda = 0.32, approx. *F* = 8.89, *p* < 0.001; Figure 4a). Separation of communities by different land‐use systems was also evident if the years 2013 (Wilks' lambda = 0.11, approx. *F* = 9.47, *p* < 0.001) and 2016 (Wilks' lambda = 0.23, approx. *F* = 14.64, *p* < 0.001) were analyzed separately (Figures A3 and A4). Species composition of Collembola communities was different in each of the land‐use systems with rainforest being more similar to oil palm than to jungle rubber. Collembola communities in jungle rubber were similar to those in rubber plantations, while rubber and oil palm plantations were distinct. Differences between land‐use systems were associated with changes in species dominance: *Isotomiella* spp. and *Pseudosinella* sp.1 were the dominant species in rainforest, *Ascocyrtus cinctus* was dominant in rubber plantations, and *Rambutsinella* cf. *scopae* was dominant in oil palm plantations (Figure A2).

Land use also significantly affected the species composition of Collembola communities in the soil layer, with the first LDA axis explaining 48% of the variation (Wilks' lambda = 0.48, approx. *F* = 5.64, *p* < 0.001; Figure 4c) which was evident in both years (Wilks' lambda = 0.23, approx. *F* = 5.37, *p* < 0.001 for 2013 and Wilks' lambda = 0.49, approx. *F* = 4.38, *p* < 0.001 for 2016) (Figures A3 and A4). *Isotomiella* spp. reached very high abundance in rainforest and was also abundant in oil palm plantations, but was less abundant in jungle rubber and rubber plantations. *Pseudosinella* sp.1 reached high abundance across all land‐use systems except for jungle rubber, and *Folsomides centralis* reached high abundance in oil palm plantations (Figure A2).

Similar to species composition, land use also strongly affected the trait composition of Collembola communities in the litter layer, with the first LDA axis explaining 53% of the variation (Wilks' lambda = 0.39, approx. *F* = 7.22, *p* < 0.001; Figure 4b), which was evident in 2013 (Wilks' lambda = 0.17, approx. *F* = 8.62, *p* < 0.001) and 2016 (Wilks' lambda = 0.36, approx. *F* = 9.14, *p* < 0.001) (Figures A3 and A4). In contrast to species community composition, trait community composition in rainforest was most distinct between rainforest and oil palm plantations, and intermediate in jungle rubber and rubber plantations. Absence of empodial appendage, sucking mouthparts, absence of furca, small body size, absence of pigmentation, and spherical abdomen were abundant traits in rainforest and jungle rubber. By contrast, long furca, present of scales, large size, patterned and diffuse pigmentation, straight furca, and presence of PAO were abundant traits in plantation systems. Details of Mahalanobis distances for species and trait community composition in the litter layer are given in Tables A7, A8 and A7, A8.

Also, trait composition of Collembola communities in the soil layer was significantly affected by land use with the first LDA axis explaining 57% of the variation (Wilks' lambda = 0.45, approx. *F* = 6.18, *p* < 0.001; Figure 4d), which was evident in both years (Wilks' lambda = 0.23, approx. *F* = 5.30, *p* < 0.001 for 2013 and Wilks' lambda = 0.45, approx. *F* = 7.29, *p* < 0.001 for 2016) (Figures A3 and A4). Trait community composition in rubber plantations was similar to that in oil palm plantations in both years (characterized by diffuse pigmentation, straight furca, presence of PAO, modified antennae, large size, and patterned pigmentation). By contrast, absent or intense pigmentation, absence of empodial appendage, small size, and spherical abdomen were more abundant traits in rainforest. Details of Mahalanobis distances for species and trait community composition in the soil layer are given in Tables A7, A8 and A7, A8.

### 3.3 Correlation between environmental factors and community composition

**FIGURE 5:**
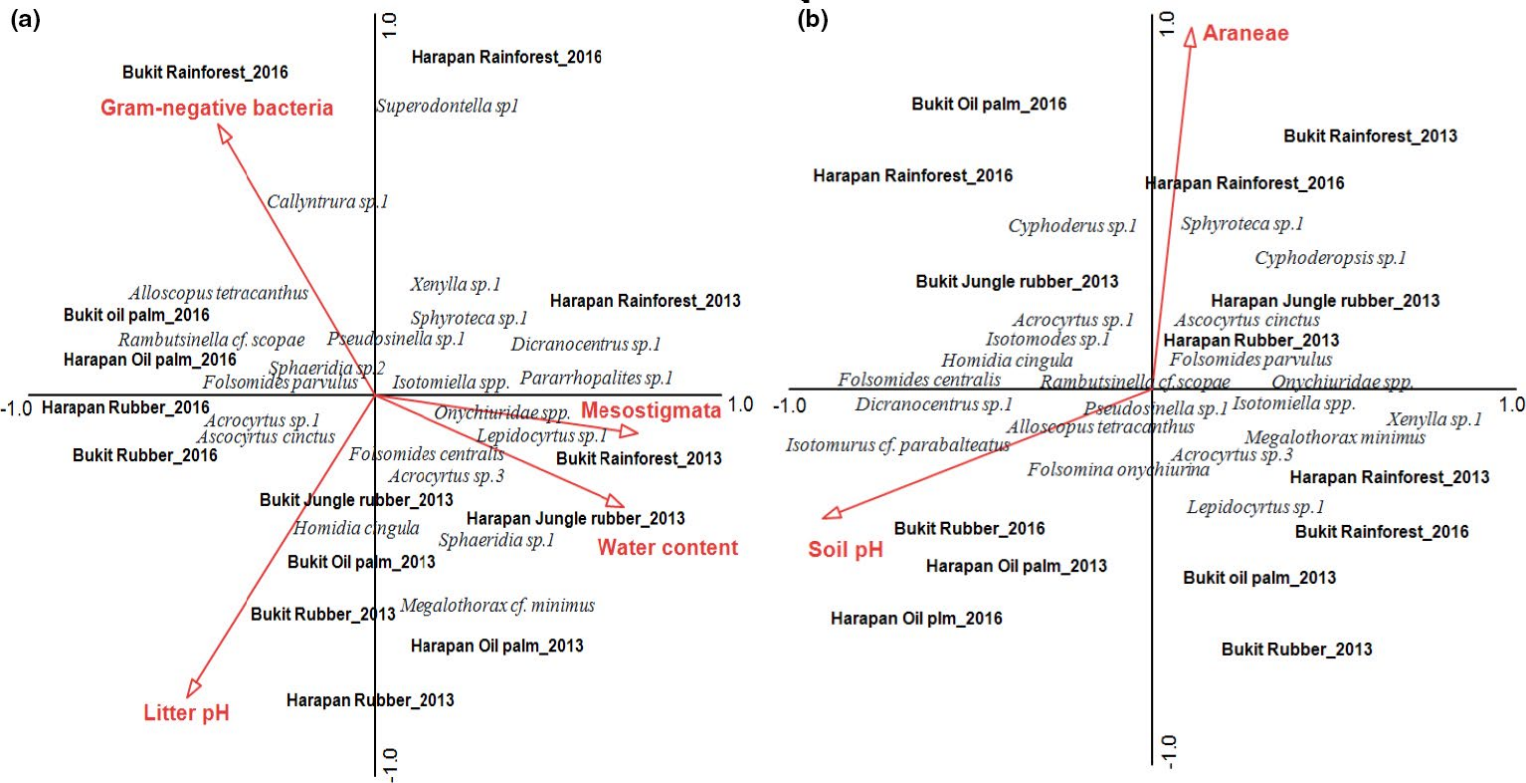
Canonical correspondence analysis of Collembola species in (a) litter and (b) soil as related to environmental variables (red arrows). Only variables selected as being significant in the forward selection procedure are shown. The four land‐use systems (rainforest, jungle rubber, rubber plantations, and oil palm plantations), 2 years (2013 and 2016), and both regions (Harapan and Bukit Duabelas) are shown together. The name of species and land‐use systems were centered to their position. The length of arrows represents the percentage variation explained by environmental variables. For environmental data, see Table A1

In the litter layer, four of the twelve environmental variables significantly correlated with species composition (CCA, forward selection), explaining 13.6% of the variation (Trace = 0.83, *F* = 1.59, *p* = 0.001; Figure 5a). Litter pH accounted for 4.2% of total variation in species composition (pseudo‐*F* = 2.5, *p* = 0.002), water content for additional 3.9% (pseudo‐*F* = 2.4, *p* = 0.003), density of Mesostigmata for 2.8% (pseudo‐*F* = 1.7, *p* = 0.033), and relative amount of Gram‐negative bacterial PLFAs for 2.8% (pseudo‐*F* = 1.8, *p* = 0.025). In 2013, rainforest and jungle rubber communities were associated with high water content and high density of Mesostigmata, whereas in 2016, rainforest communities were associated with high amount of Gram‐negative bacteria only. Communities in rubber and oil palm plantations in both 2013 and 2016 were associated with high litter pH. Several atmobiotic and epedaphic species, that is, *Acrocyrtus* sp.3, *Lepidocyrtus* sp.1, *Pararrhopalites* sp.1, *Sphaeridia* sp.1, and *Sphaeridia* sp.2, but also euedaphic species, that is, *Pseudosinella* sp.1, *Isotomiella* spp., and *Onychiuridae* spp., were associated with high water content and density of Mesostigmata, whereas *Superodontella* sp.1, *Xenylla* sp.1, and *Callyntrura* sp.1 were associated with Gram‐negative bacteria. *Ascocyrtus cinctus*, *Rambutsinella* cf. *scopae*, *Folsomides parvulus*, *Folsomides centralis*, *Homidia cingula*, *Acrocyrtus* sp.1, and *Megalothorax* cf. *minimus* in plantation systems were associated with high litter pH.

In the soil layer, only two of the twelve environmental variables significantly correlated with species composition, together explaining 5.7% of the variation (Trace = 0.67, *F* = 1.27, *p* = 0.02; Figure 5b). Based on forward selection, density of Araneae accounted for 2.9% of the total variation in community composition (pseudo‐*F* = 1.7, *p* = 0.044) and soil pH accounted for additional 2.8% (pseudo‐*F* = 1.7, *p* = 0.045). In line with the effects of environmental factors in the litter layer, in the soil layer high pH was associated with high density of several atmobiotic species, that is, *Homidia cingula*, *Isotomurus* cf. *parabalteatus*, *Dicranocentrus* sp.1, and *Acrocyrtus* sp.1. By contrast, *Pseudosinella* sp.1, *Isotomiella* spp., *Sphyrotheca* sp.1, *Cyphoderopsis* sp.1, *Xenylla* sp.1, and *Folsomides parvulus* were associated with high density of Araneae (potential macropredator) in rainforest.

**FIGURE 6:**
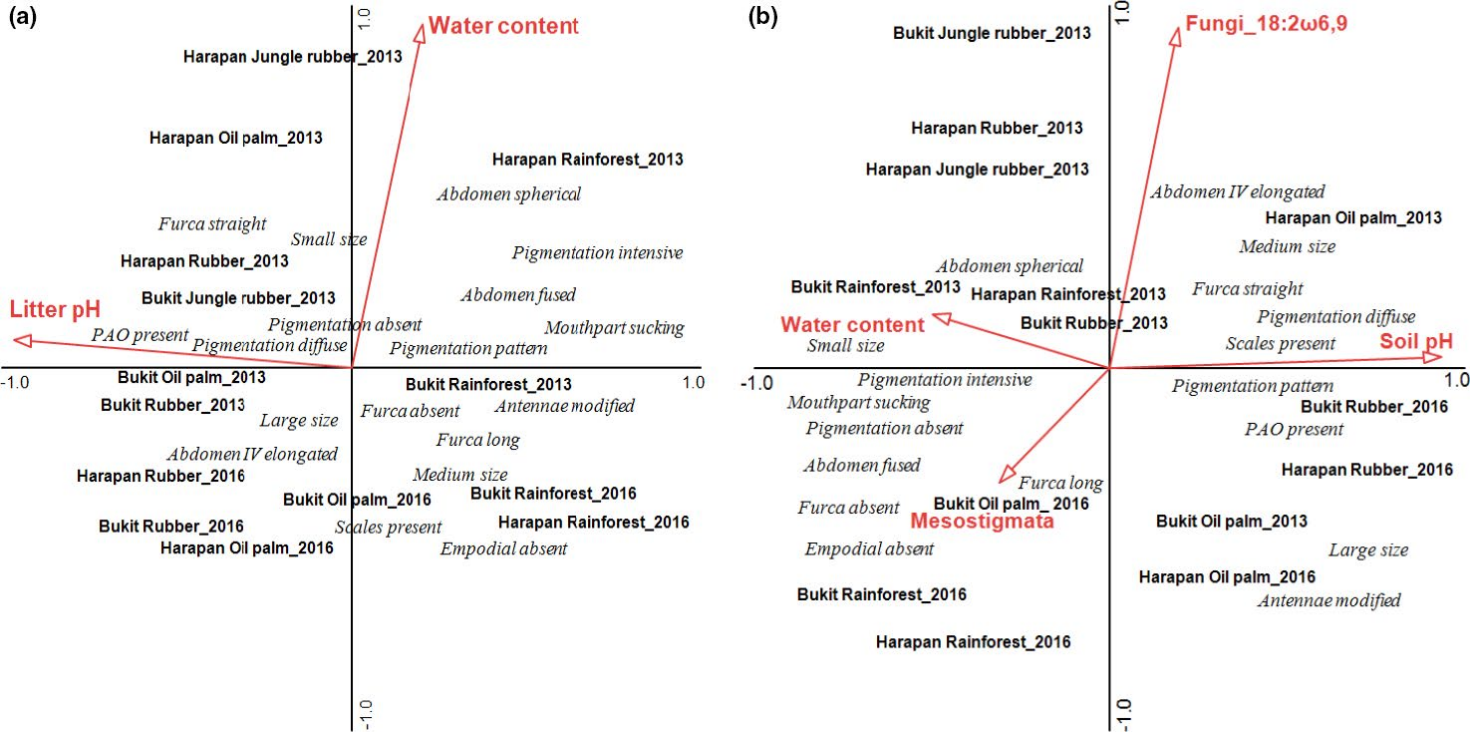
Redundancy analysis of Collembola traits in (a) litter and (b) soil as related to environmental variables (red arrows). Only variables selected as being significant during the forward selection procedure are shown. All land‐use systems (rainforest, jungle rubber, rubber plantations, and oil palm plantations), 2 years (2013 and 2016), and both regions (Harapan and Bukit Duabelas) are shown together. The traits and land‐use systems were centered to their position. The length of arrows represents the percentage variation explained by environmental variables. For environmental data, see Table A1

In the litter layer, none of the environmental variables were correlated significantly with the trait composition of Collembola communities (RDA, forward selection; Trace = 0.22, *F* = 1.09, *p* = 0.27; Figure 6a). While the overall model was not significant, both water content and pH were identified as significant factors, accounting for 3.6 and 3.3% of the total variation in trait composition, respectively (pseudo‐*F* = 2.2, *p* = 0.045 and pseudo‐*F* = 2.1, *p* = 0.048, respectively).

In the soil layer, four of the twelve environmental variables were significant, explaining 15.2% of the variation in the trait composition (Trace = 0.26, *F* = 1.39, *p* = 0.02; Figure 6b). Based on forward selection, water content accounted for 4.4% of total variation in trait composition (pseudo‐*F* = 2.7, *p* = 0.022), soil pH for 3.9% (pseudo‐*F* = 2.5, *p* = 0.012), abundance of Mesostigmata for 3.4% (pseudo‐*F* = 2.2, *p* = 0.026), and the fungal PLFA marker (18:2ω6,9) for 3.4% (pseudo‐*F* = 2.2, *p* = 0.014). High pH values were correlated positively with diffuse pigmentation, straight furca, presence of PAO, presence of scales, modified antennae, large body size, and patterned pigmentation. High water content and to some extent the fungal PLFA marker were correlated positively with absence of furca, small body size, spherical and fused abdomen, absent and intensive pigmentation, and sucking mouthparts. In addition, high density of Mesostigmata was correlated positively with long furca and absence of empodial appendage.

## 4 DISCUSSION

Our study comprehensively assessed the response of the species and trait composition of Collembola communities in litter and soil after conversion of rainforest into plantation systems. The results indicate that changes on density, species richness, and indices of functional diversity of Collembola vary moderately among the studied land‐use systems as response to the conversion of rainforest and environmental changes (Deharveng, 1996; Devi et al., 2011; Rusek, 1998; Sousa et al., 2006). However, density, species richness, and functional diversity (FD) consistently declined in the litter layer of rubber and especially oil palm plantations. Despite the only moderate changes in community metrics, trait and species composition of Collembola communities were distinct in all land‐use systems studied. Even in the extensively managed jungle rubber agroforesty system, species and trait composition of Collembola communities differed clearly from that in rainforest. Among the studied environmental variables, Collembola community composition was related closest to pH and water content with correlations between environmental factors and changes in communities being more pronounced in litter than in soil.

### 4.1 Density, species richness, functional diversity, and functional dispersion

In part supporting our first hypothesis, density, species richness and FD of Collembola were at a maximum in litter of rainforest. This is in line with the study of Martius et al. (2004) in central Amazonia reporting more Collembola species in rainforest than in rubber and peach palm plantations. In another study from Jambi Province, density and species richness of soil Collembola decreased gradually from rainforest to jungle rubber to rubber plantations (Deharveng, 1992 in Michon & de Foresta, 1995). This was expected since there is less litter (Krashevska et al., 2015) and thus less space and food for Collembola in rubber and even less in oil palm plantations. The amount of litter is likely to be the main factor driving Collembola communities since, according to our results, the density of Collembola per gram of carbon in the litter was similar across all land‐use systems. In trend, species richness, FD, and FDis also were lowest in the litter layer of oil palm plantations. This suggests that in intensively managed plantations, mulching practices may help in increasing the density and species richness of Collembola and ecosystem processes Collembola are involved in (Tao et al., 2018).

In contrast to our first hypothesis, density, species richness, and diversity indices of Collembola were similar or even slightly higher in soil of oil palm plantations than in soil of rainforest. This suggests that the soil in plantations provides suitable environmental conditions for Collembola allowing a variety of species to survive and successfully reproduce even in monoculture plantations of oil palm as well as rubber. The land‐use effect was least pronounced for the total community FDis, suggesting that species with different functional traits may reach high densities in monoculture plantations. The results are in contrast to the study of Winck et al. (2017) in southern Brazil, which found that functional diversity of Collembola was significantly higher in tropical forest than in grassland and plantation systems. High density of soil Collembola in plantations may be related to the buffering function of the soil against adverse environmental conditions, thereby allowing Collembola to cope with disturbances (Filser, 1995). For the temperate zone, it has been shown that Collembola may reach high population densities even in intensively managed agro‐ecosystems (Chauvat, Ponge, et al., 2007; Chauvat, Wolters, et al., 2007; Filser, 1995; Frampton & Brink, 2002). In Borneo, the conversion of rainforest into oil palm plantations reduced species richness in a large number of taxa except in scavenging mammals and Collembola (Edwards et al., 2014). In general, soil food webs in oil palm plantations are not limited by the available energy in comparison with rainforest (Potapov et al., 2019). Fertilization and weeding likely provide additional resources for the detrital system in plantations (Clough et al., 2016). Further, Susanti et al. (2019) reported that algae serve as important food resource for Collembola in tropical ecosystems, especially in oil palm plantations. Schulz et al. (2019) reported the density of photoautotrophic protists to be strongly increased in oil palm plantations compared to rainforest at our study sites. In addition, it is increasingly recognized that Collembola benefit from root‐based resources in both forests and agricultural systems (Fujii et al., 2014; Li et al., 2020; Potapov et al., 2016) and this likely contributed to the high density and number of species of Collembola in plantations. Another explanation for the high density of Collembola in soil of oil palm plantations may be reduced top‐down control by predators. In fact, the density of small soil‐dwelling spiders, one of the main Collembola predators (Lawrence & Wise, 2000), is low in plantations (Potapov et al., 2020).

### 4.2 Species and trait community composition

Supporting in part our second hypothesis, communities of Collembola differed among all the four land‐use systems studied. However, in contrast to our expectations community composition in jungle rubber and rainforest was distinctly different, especially in the litter layer. Further, unexpectedly, a number of Collembola species present in rainforest were also present in monoculture plantations. The community core included *Isotomiella* spp. and *Pseudosinella* sp.1 in rainforest, which were replaced by *Folsomides* spp. in jungle rubber and monoculture plantations, suggesting a uniform change in community composition already starting in extensively managed agroforestry systems, that is, jungle rubber. *Folsomides* spp. are pantropical parthenogenetic species, usually numerically dominant in soils of disturbed ecosystems all over the tropics. Our study shows that they may reach high density in disturbed habitats even if the disturbance level is low. The observed shift in community composition is also in line with strong changes in energy channeling in the belowground food web after conversion of rainforest into agricultural land‐use systems at our study sites, that is, increased energy sequestration in large decomposers and decreased predation (Potapov et al., 2019). This highlights the susceptibility of the belowground system to changes in land use.

Confirming our third hypothesis, water content and pH were the most important environmental factors explaining species and trait composition of Collembola in both litter and soil. Generally, effects of environmental factors were more pronounced in litter than in soil, presumably because soil buffers variations in environmental conditions (Cassagnau, 1961), whereas biota in the litter layer are more heavily exposed to such variations (Krashevska et al., 2015, 2019). The strong effect of pH in structuring the species and trait composition of Collembola likely is related to the increase in soil pH with the conversion of rainforest into plantation systems and associated changes in microbial community composition (Berkelmann et al., 2020; Brinkmann et al., 2019). The functional link between pH and changes in trait composition, however, remains to be discovered. Moreover, most of the variation in Collembola community composition in litter and soil remained unexplained suggesting that stochastic or spatial processes were of major importance.

Species composition in litter in rainforest was associated with high water content and high density of Mesostigmata and Gram‐negative bacteria. The relevance of water content for Collembola community composition is in line with a number of studies from temperate regions (Fujii et al., 2014; Kuznetsova, 2003; Ponge, 1993). Soil moisture controls the risk of desiccation and the availability of microorganisms as food for Collembola (Fierer et al., 2003; Rousk et al., 2010; Zhang et al., 2013). High amounts of organic matter and high water holding capacity in rainforest soil likely improve habitat conditions and increase the availability of food resources in particular for euedaphic Collembola (Muturi et al., 2011). The presence of a number of species, including *Acrocyrtus* sp.3, *Lepidocyrtus* sp.1, *Pararrhopalites* sp.1, *Sphaeridia* sp.1, *Sphaeridia* sp.2, and two euedaphic species (*Pseudosinella* sp.1 and *Onychiuridae* spp.) in rainforest, as well as in jungle rubber, was associated with high water content. Further, the density of some Collembola species in litter of rainforest also correlated with the amount of Gram‐negative bacteria, especially two Poduromorpha species, that is, *Xenylla* sp.1 and *Superodontella* sp.1, suggesting that these species may feed on bacteria or bacterial feeding nematodes. Species in litter and soil of plantations, such as *Homidia cingula*, *Isotomurus* cf. *parabalteatus*, *Dicranocentrus* sp.1, *Acrocyrtus* sp.1, *Folsomides centralis*, and *Megalothorax* cf. *minimus*, were associated with high pH. In litter, however, high pH also was associated with low concentrations of Gram‐negative bacteria.

Salmon et al. (2014) showed that traits of species living in open habitats and adapted to light exposure and dry conditions included high mobility (long furca), large body size, presence of scales, pigmentation (as protection against UV), and organs for sensing wind and light. Further, Winck et al. (2017) reported that open habitat is linked to sensorial traits (number of ocelli, antenna length, and trichobothria) and drought tolerance traits (body size). Salmon and Ponge (2012) also showed that large mobile species were associated with agricultural systems with low amounts of litter. All these traits of large mobile species were also associated with plantations in our study. Presence of more Collembola with patterned coloration in plantations in comparison with rainforest was in line with more pigmented ground spiders at these sites (Potapov et al., 2020). Presumably, coloration patterns in ground‐dwelling arthropods (epedaphic Collembola and spiders) function as camouflage in rubber and oil palm plantations with more open canopies and shallow litter layer. Recording such universal trait patterns across groups may help linking traits and environmental conditions in the future. However, more data are needed to achieve this goal. For example, in our study Collembola species with PAO reached high density in plantations, whereas the density of Collembola species with PAO was found to be low in agricultural sites in the temperate region (Salmon & Ponge, 2012; Salmon et al., 2014). In our study, Collembola traits correlated with water content and soil pH, whereas this was not the case in the study of Salmon and Ponge (2012), suggesting that trait–environment relationships may differ between temperate and tropical ecosystems or between different types of soils.

Finally, in contrast to our fourth hypothesis, species composition varied in a more predictable way with environmental factors than trait composition, particularly in the litter layer, where environmental factors did not significantly influence the trait composition of Collembola. Due to the scarcity of information on the biology of tropical Collembola, we focused on morphological traits, which we assumed to be functional (Vandewalle et al., 2010). More detailed information on functional traits, including biological, physiological, and morphological traits, is needed for understanding trait–environment relationships in Collembola (Raymond‐Léonard et al., 2019). On the other hand, the environmental factors included in this study may have missed important drivers of Collembola community composition. For example, the community composition of Collembola in rubber plantations differed from that of the other studied land‐use systems, but the environmental factors studied failed to explain this difference. Hence, while supporting the usefulness of the trait‐based approach, our analysis also highlighted the need for more information on functional traits of Collembola in tropical regions.

### 4.3 Dominant tropical Collembola

Dominating species at our study sites were previously recorded across various tropical regions, suggesting that certain Collembola species/taxa are well adapted to and widespread across tropical ecosystems. Collembola communities were dominated by species of the family Isotomidae, particularly the genera *Folsomides* and *Isotomiella* (43% of total individuals). This is in line with the study of Warino et al. (2017) in Jambi Province who reported that *Folsomides* and *Isotomiella* may represent 40% of total Collembola density in plantation systems. In tropical montane forests of the Doi Inthanon in Thailand, Deharveng et al. (1989) also found *Isotomiella* spp. among the dominant species, in particular in organic layers. Similarly, Muturi et al. (2011) reported *Isotomiella* species to be among the most abundant Collembola in intensively managed agricultural systems in Kenya. Several species of this genus with similar morphology have been found in Sumatra (Deharveng & Suhardjono, 1994), but their ecology is little known. Three of them are particularly abundant in Sumatra (*I. symetrimucronata* Najt and Thibaud, 1987, the most abundant, *I. nummulifer* Deharveng and Oliveira, 1990, and *I. cribrata* Deharveng & Suhardjono, 1994). Further information on *Isotomiella* species in different land‐use systems is needed to allow insight into the pantropical diversification of this genus.

The other dominant genus of Isotomidae at our study sites was *Folsomides*, with the species *F. centralis* and *F. parvulus*. These two species are also codominant in many tropical regions across the world, with the latter especially abundant in disturbed habitats (Deharveng et al., 2020). At our study sites, *F. centralis* dominated in each of the plantations, confirming its preference for disturbed habitats. Similarly, in the tropical region of Oaxaca (Mexico) Rojas et al. (2009) reported *Folsomina onychiurina* to dominate in disturbed systems. At our study sites, *F. onychiurina* was also present but it was rare. An important characteristic of these pantropical Isotomidae is their mode of reproduction: All species stated above are parthenogenetic, which may explain their large distribution across the world, as well as their capacity to colonize disturbed habitats.

Besides Isotomidae, species of the family Entomobryidae reached high density in soil in most land‐use systems. *Pseudosinella* sp.1 reached high abundance (30%), reflecting that this species also is well adapted to the environmental conditions in agro‐ecosystems. This is in line with the study of Rojas et al. (2009) reporting that *Pseudosinella* sp. may reach high density in fallow soils. The records of another Entomobryidae species, *Ascocyrtus cinctus*, from our study sites also fit earlier studies from Sumatra (Fatimah et al., 2012; Selvany, 2018).

Collembola communities across contrasting land‐use systems were numerically dominated by small, white, or pale‐colored species, often lacking eyes. However, true euedaphic (i.e., soil‐dwelling) species of the families Onychiuridae and Tullbergiidae, typically abundant in temperate ecosystems, were rare in our study region, presumably due to biogeographical history rather than current ecological conditions (Deharveng et al., 1989). The similarity of Collembola communities across tropical regions suggests that several abundant pantropical species dominate in many natural and transformed tropical ecosystems, either due to historical reasons or adaptation to high temperature and other climate characteristics. Different preferences observed in pantropical genera and species of Collembola call for more focused studies on their genetic diversity and ecological preferences across tropical regions to reveal the history and diversification of Collembola across the tropics and to understand their contribution to soil community functioning.

## 5 CONCLUSIONS

Overall, changes in density, species richness, and diversity indices of Collembola communities with conversion of rainforest into plantation systems were moderate and were more pronounced in the litter than the soil layer. Collembola density and species richness were higher in litter than in soil in rainforest, whereas in oil palm plantations, it was the opposite. Community composition of Collembola changed strongly with rainforest conversion, but certain pantropical species were present in high numbers across the studied land‐use systems, suggesting that these species are resistant to rainforest conversion and associated changes in environmental factors. Unexpectedly, the community composition of Collembola differed to a similar extent between rainforest, jungle rubber, and monoculture plantations suggesting that tropical Collembola communities are sensitive to even moderate changes in land use. Collembola communities in plantations were characterized by high abundance of species with sensory‐ and mobility‐associated traits, suggesting that such traits help soil‐dwelling arthropods in colonizing plantations. Water content and pH were identified as environmental factors associated with species and trait composition of Collembola communities across land‐use systems and layers. Further, Collembola communities likely were also structured by top‐down control by micro‐ and macropredators (gamasid mites and spiders). However, environmental factors explained only up to 13.6% of Collembola community composition, suggesting that factors not included in this study may be more important. Overall, conversion of rainforest into plantation systems altered the composition of Collembola communities with potential consequences for decomposition processes and other ecosystem services they provide. To better understand the community assembly and functioning of tropical Collembola communities, studies on the ecology and genetic diversity of pantropical genera are needed.

## CONFLICT OF INTEREST

The authors declare that they have no conflict of interest.

## AUTHOR CONTRIBUTIONS

**Winda Ika Susanti:** Formal analysis (equal); Visualization (equal); Writing‐original draft (equal); Writing‐review & editing (equal). **Tamara Bartels:** Formal analysis (equal). **Valentyna Krashevska:** Data curation (equal); Formal analysis (equal); Visualization (equal). **Rahayu Widyastuti:** Project administration (equal). **Louis Deharveng:** Supervision (equal); Writing‐review & editing (equal). **Stefan Scheu:** Conceptualization (equal); Funding acquisition (equal); Investigation (equal); Project administration (equal); Supervision (equal); Validation (equal); Writing‐review & editing (equal). **Anton Potapov:** Conceptualization (equal); Data curation (equal); Investigation (equal); Methodology (equal); Supervision (equal); Writing‐review & editing (equal).

## CONTRIBUTION TO THE FIELD STATEMENT

Collembola are major components of the diversity of soil invertebrates and useful indicators of changes in soil conditions and environmental factors, particularly in response to rainforest conversion into agricultural plantations, a hot topic in tropical regions. The study presents the first detailed species‐level assessment of the differences in species and trait composition of Collembola communities between rainforest and monoculture plantations of rubber and oil palm in the tropics. The results document that the conversion of rainforest into plantation systems alters the functional composition of Collembolan communities with potential consequences for decomposition processes and other ecosystem services they provide. However, the results also show that some species are resistant to rainforest conversion and associated changes in environmental factors. Overall, the study presents community and trait composition of Collembola as valuable approach to investigate effects of rainforest conversion on soil biodiversity and ecosystem functioning.

## Supporting information

Supplementary Material

## Figures and Tables

**FIGURE A1:**
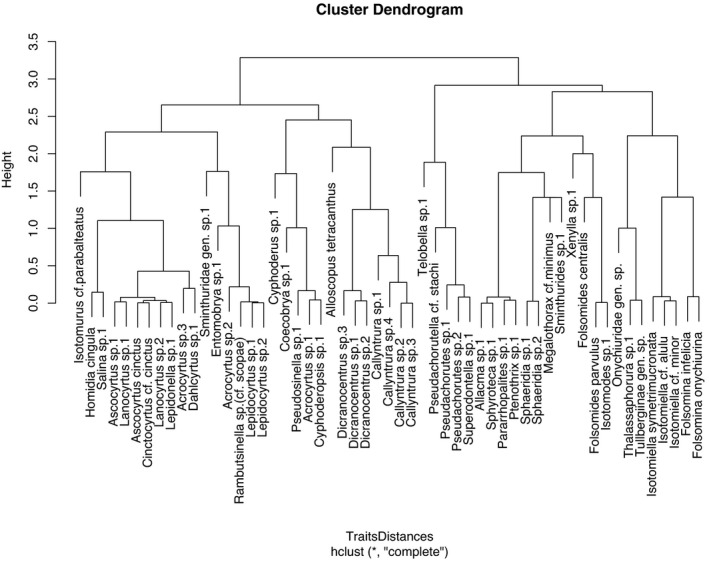
Hierarchical clustering of species based on their trait values

## Data Availability

Collembola species and trait matrix data and environmental data analysis are available at https://doi.org/10.5061/dryad.f4qrfj6vj.

## APPENDIX A

**TABLE A1:** Mean values of environmental factors in the litter and soil layer in the four land‐use systems studied (rainforest, jungle rubber, rubber, and oil palm plantations) in the years 2013 and 2016

	Year	Water content (%)	pH	C‐to‐N ratio	NLFA 16:1ω5c (c nmol/g)	PLFA 18:2ω6,9 (%)	PLFA 20:5ω3 (%)	Gr^+^ bacteria (%)	Gr^−^ bacteria (%)	Araneae log10 (ind/sample)	Formicidae log10 (ind/sample)	Mesostigmata log10 (ind/sample)	Oribatida log10 (ind/sample)
Litter layer													
Rainforest	2013 (*n* = 8)	184.0	4.3	31.9	6.7	18.4	0.7	33.8	30.4	0.32	0.73	1.53	2.09
Rainforest	2016 (*n* = 11)	71.61	3.8	25.1	11.4	17.7	1.4	46.3	40.2	0.19	0.37	1.13	1.91
Jungle rubber	2013 (*n* = 8)	198.0	5.1	27.1	21.4	18.1	0.3	36.4	28.2	0.07	0.13	1.10	2.03
Oil palm	2013 (*n* = 7)	149.4	5.4	29.4	13.9	17.4	0.3	37.5	27.9	0.04	0.15	0.99	1.56
Oil palm	2016 (*n* = 11)	35.3	5.1	22.1	7.2	15.2	1.4	46.9	37.6	0	0	0.82	1.32
Rubber	2013 (*n* = 7)	116.1	5.5	29.5	15.1	20.7	0.7	39.9	24.8	0	0.51	1.07	1.72
Rubber	2016 (*n* = 11)	41.0	5.3	26.7	8.3	20.8	1.4	40.5	38.6	0.08	0.22	0.85	1.28
Soil layer													
Rainforest	2013 (*n* = 8)	71.7	3.7	15.1	7.3	7.8	0.3	58.4	37.8	0.35	1.03	0.81	1.08
Rainforest	2016 (*n* = 12)	73.6	3.9	14.1	5.9	5.7	0.9	68.4	43.8	0.33	0.82	0.64	0.91
Jungle rubber	2013 (*n* = 8)	95.5	4.3	17.0	15.0	10.2	0.3	50.2	24.3	0.32	0.39	0.56	1.11
Oil palm	2013 (*n* = 8)	77.4	4.7	13.4	18.6	8.1	0.1	55.1	30.6	0.04	0.25	0.83	1.58
Oil palm	2016 (*n* = 10)	43.3	4.9	12.	18.8	6.7	0.3	72.1	29.1	0.19	0.62	1.14	1.24
Rubber	2013 (*n* = 8)	59.0	4.3	11.9	13.9	8.8	0.1	45.5	21.7	0.13	0.59	0.88	1.38
Rubber	2016 (*n* = 12)	39.8	5.0	14.5	5.9	7.7	0.5	65.1	30.7	0.29	0.52	0.58	0.81

Note

PLFA 18:2ω6,9, PLFA 20:5ω3, Gr^+^ bacteria markers (i15:0, a15:0, i16:0, i17:0), and Gr^−^ bacteria markers (2OH 12:0, 2OH 14:0, 16:1ω7, cy17:0, 2OH 16:0, cy19:0, 2OH 10:0) were as results from arcsine square root transformation; Araneae, Formicidae, Mesostigmata, and Oribatida were as results from log transformation; and *n* represents the number of plot in each year.

**TABLE A2:** Selected model and results for density based on region, system, layer, plot, and year

Model: Variable ~ 1 + (Layer + System)^2 + Landscape + (1|YearPlot)							
*Df*	AIC	BIC	logLik	Deviance	Chisq	Chi *Df*	Pr(>Chisq)
11	1,360.1	1,391.9	−669.0	1,338.1	2.0	0	0.0000

Results of the selected model for density per area			
	Chisq	*Df*	Pr(>Chisq)
Layer	2.7	1	0.1025
System	6.7	3	0.0833
Landscape	3.5	1	0.0627
Layer:System	11.8	3	0.0081

Results of Tukey test for density of system and layer				
System	Layer	Mean	*SD*	Letter
Jungle Rubber	Litter	73.5	56.8	ab
Jungle Rubber	Soil	32.6	22.0	ab
Oil Palm	Litter	36.2	65.7	b
Oil Palm	Soil	77.4	51.5	a
Rainforest	Litter	71.0	61.2	ab
Rainforest	Soil	84.0	124.5	ab
Rubber	Litter	38.7	31.6	ab
Rubber	Soil	47.7	23.9	ab

**TABLE A3:** Selected model and results for species richness based on landscape, system, layer, plot, and year

Model: Variable ~ 1 + (Layer + System)^2 + Year + (1|YearPlot)							
*Df*	AIC	BIC	logLik	Deviance	Chisq	Chi *Df*	Pr(>Chisq)
11	654.4	686.2	−316.2	632.4	35.5	0	0.0000

Results of the selected model for species richness			
	Chisq	*Df*	Pr(>Chisq)
Layer	1.4	1	0.2297
System	7.5	3	0.0588
Year	48.7	1	0.0000
Layer:System	14.5	3	0.0023

Results of Tukey test for species richness of system and layer					
System	Layer	Mean		*SD*	Letter
Junggle Rubber	Litter	6.75		2.71	ab
Junggle Rubber	Soil	6.00		3.38	ab
Oil Palm	Litter	3.68		2.76	b
Oil Palm	Soil	6.68		3.57	a
Rainforest	Litter	6.95		5.14	a
Rainforest	Soil	5.35		3.09	ab
Rubber	Litter	4.30		2.71	ab
Rubber	Soil	5.10		2.22	ab

**TABLE A4:** Selected model and results for functional diversity based on landscape, system, layer, plot, and year

Model:Variable ~ 1 + (Layer + System)^2 + Year + Riparian + Landscape + (1|YearPlot)							
*Df*	AIC	BIC	logLik	Deviance	Chisq	Chi *Df*	Pr(>Chisq)
13	826.5	863.7	−400.3	800.5	32.2	2	0.0000

Result of the selected model for functional diversity			
	Chisq	*Df*	Pr(>Chisq)
Layer	2.9	1	0.0887
System	6.4	3	0.0933
Year	29.4	1	0.0000
Riparian	0.1	1	0.7876
Landscape	3.6	1	0.0570
Layer:System	14.0	3	0.0029

Results of Tukey test for functional diversity of system and layer				
System	Layer	Mean	*SD*	Letter
Junggle Rubber	Litter	14.10	4.41	ac
Junggle Rubber	Soil	17.10	6.13	ac
Oil Palm	Litter	9.60	6.23	c
Oil Palm	Soil	15.81	7.01	a
Rainforest	Litter	16.32	8.91	ab
Rainforest	Soil	12.81	6.91	ac
Rubber	Litter	10.20	5.90	bc
Rubber	Soil	12.11	4.51	ac

**TABLE A5:** Selected model and results for density per gram carbon based on region, system, layer, plot, and year

Model: Variable ~ 1 + Layer + System)^2 + Riparian + (1|Year) + (1|Year/Plot) + (1|Landscape)							
*Df*	AIC	BIC	logLik	Deviance	Chisq	Chi *Df*	Pr(>Chisq)
11	1,275.7	1,307.5	626.8	1,253.7	6.7	0	0.0000

Result of the selected model for density per gram of carbon			
	Chisq	*Df*	Pr(>Chisq)
Layer	73.6	1	0.0000
System	4.5	3	0.2089
Year	7.5	1	0.0061
Layer:System	10.3	3	0.0162

Results of Tukey test for density per gram carbon of system and layer				
System	Layer	Mean	*SD*	Letter
Junggle Rubber	Litter	9.11	7.22	a
Junggle Rubber	Soil	0.57	0.32	d
Oil Palm	Litter	5.75	5.94	a
Oil Palm	Soil	1.70	1.13	bc
Rainforest	Litter	4.22	3.47	ab
Rainforest	Soil	1.42	1.99	cd
Rubber	Litter	4.19	3.60	ab
Rubber	Soil	1.09	0.57	cd

**TABLE A6:** Selected model and results for functional dispersion based on region, system, layer, plot, and year

Model: Variable ~ 1 + (Layer+System)^2 + Year + (1|YearPlot)							
*Df*	AIC	BIC	logLik	Deviance	Chisq	Chi *Df*	Pr(>Chisq)
11	−341.6	−310.2	181.8	−363.6	3.80	2	0.00

Results of the selected model for functional dispersion			
	Chisq	*Df*	Pr(>Chisq)
Layer	0.1	1	0.8981
System	1.1	3	0.7601
Year	3.8	1	0.0510
Layer:System	12.6	3	0.0053

**TABLE A7:** Mahalanobis distances from LDA of species composition in litter and soil layer

	*F*‐value	*df*1	*df*2	*p*‐value	MD^2^
Litter layer					
Rainforest–Jungle rubber	6.57	3	22	0.0024	3.2315
Rainforest–Rubber	14.87	3	32	0.0001	1.4898
Rainforest–Oil palm	4.73	3	31	0.0078	3.1200
Jungle rubber–Rubber	4.50	3	22	0.0130	1.6731
Jungle rubber–Oil palm	5.35	3	21	0.0067	1.9256
Rubber–Oil palm	NaN	3	14	NaN	2.5382
Soil layer					
Rainforest–Jungle rubber	5.18	3	23	0.0069	2.2691
Rainforest–Rubber	8.00	3	36	0.0003	1.7148
Rainforest–Oil palm	9.30	3	33	0.0001	1.8029
Jungle rubber–Rubber	3.08	3	23	0.0471	1.4614
Jungle rubber–Oil palm	3.00	3	20	0.0544	1.7851
Rubber–Oil palm	NA	3	16	NA	1.6884

**TABLE A8:** Mahalanobis distances from LDA of trait composition in litter and soil layer

	*F*‐value	*df*1	*df*2	*p*‐value	MD^2^
Litter layer					
Rainforest–Jungle rubber	4.74	3	22	0.0106	2.2898
Rainforest–Rubber	5.73	3	32	0.0029	2.5403
Rainforest–Oil palm	9.99	3	32	0.0001	1.7000
Jungle rubber–Rubber	4.62	3	22	0.0117	2.4590
Jungle rubber–Oil palm	4.53	3	22	0.0127	2.5188
Rubber–Oil palm	NA	3	14	NA	1.8473
Soil layer					
Rainforest–Jungle rubber	6.00	3	23	0.0035	3.2876
Rainforest–Rubber	9.35	3	36	0.0001	1.8002
Rainforest–Oil palm	7.90	3	33	0.0004	1.9245
Jungle rubber–Rubber	4.13	3	23	0.0175	2.2041
Jungle rubber–Oil palm	7.63	3	20	0.0013	2.3269
Rubber–Oil palm	NA	3	16	NA	1.0510

## References

[ece37881-bib-0001] Adams, E. C. G. (1979). ‘On the feeding methods and fine structure of the mouth‐parts of *Ceratrimeria leleupi* Salmon and Adams (Collembola: Neanuridae). Zoology Publication from Victoria University of Wellington (72).

[ece37881-bib-0002] Allen, K. , Corre, M. D. , Tjoa, A. , & Veldkamp, E. (2015). Soil nitrogen‐cycling responses to conversion of lowland forests to oil palm and rubber plantations in Sumatra, Indonesia. PLoS One, 10(7), 1–21. 10.1371/journal.pone.0133325 PMC4519237 26222690

[ece37881-bib-0003] Barnes, A. D. , Jochum, M. , Mumme, S. , Haneda, N. K. , Farajallah, A. , Widarto, T. H. , & Brose, U. (2014). Consequences of tropical land use for multitrophic biodiversity and ecosystem functioning. Nature Communications, 5, 1–7. 10.1038/ncomms6351 PMC4220457 25350947

[ece37881-bib-0004] Bates, D. , Maechler, M. , Bolker, B. , & Walker, S. (2015). Fitting linear mixed‐effects model using lme4. Journal of Statistical Software, 67(1), 1–48. 10.18637/jss.v067.i01

[ece37881-bib-0005] Berkelmann, D. , Schneider, D. , Meryandini, A. , & Daniel, R. (2020). Unravelling the effects of tropical land use conversion on the soil microbiome. Environmental Microbiome, 15, 5. 10.1186/s40793-020-0353-3 33902736 PMC8067294

[ece37881-bib-0006] Brinkmann, N. , Schneider, D. , Sahner, J. , Ballauff, J. , Edy, N. , Barus, H. , Irawan, B. , Budi, S. W. , Qaim, M. , Daniel, R. , & Polle, A. (2019). Intensive tropical land use massively shifts soil fungal communities. Scientific Report, 9(1), 3403. 10.1038/s41598-019-39829-4 PMC6399230 30833601

[ece37881-bib-0007] Cassagnau, P. (1961). Ecologie du sol dans les Pyrénées Centrales. Les biocénoses de Collemboles (p. 235). Hermann.

[ece37881-bib-0008] Chagnon, M. , Pare, D. , Hebert, C. , & Camire, C. (2001). Effects of experimental liming on collembolan communities and soil microbial biomass in a southern Quebec sugar maple (*Acer saccharum* Marsh.) stand. Applied Soil Ecology, 17(1), 81–90. 10.1016/S0929-1393(00)00134-7

[ece37881-bib-0009] Chauvat, M. , Ponge, J. F. , & Wolters, V. (2007). Humus structure during a spruce forest rotation: Quantitative changes and relationship to soil biota. European Journal of Soil Science, 58(3), 625–631. 10.1111/j.1365-2389.2006.00847.x

[ece37881-bib-0010] Chauvat, M. , Wolters, V. , & Dauber, J. (2007). Response of collembolan communities to land‐use change and grassland succession. Ecography, 30(2), 183–192. 10.1111/j.0906-7590.2007.04888.x

[ece37881-bib-0012] Christiansen, K. A. (1965). Behavior and form in the evolution of cave Collembola. Evolution, 19(4), 529–537. 10.1111/j.1558-5646.1965.tb03328.x

[ece37881-bib-0013] Clough, Y. , Krishna, V. V. , Corre, M. D. , Darras, K. , Denmead, L. H. , Meijide, A. , Moser, S. , Musshoff, O. , Steinebach, S. , Veldkamp, E. , Allen, K. , Barnes, A. D. , Breidenbach, N. , Brose, U. , Buchori, D. , Daniel, R. , Finkeldey, R. , Harahap, I. , Hertel, D. , … Scheu, S. (2016). Land‐use choices follow profitability at the expense of ecological functions in Indonesian smallholder landscapes. Nature Communications, 7, 13137. 10.1038/ncomms13137 PMC5062595 27725673

[ece37881-bib-0014] Coulibaly, S. F. M. , Winck, B. , Akpa‐Vinceslas, M. , Mignot, L. , Le Gras, M. , Forey, E. , & Chauvat, M. (2019). Functional assemblages of Collembola determine soil microbial communities and associated functions. Frontiers in Environmental Science, 7, 52. 10.3389/fenvs.2019.00052

[ece37881-bib-0016] Daróczi, G. , & Tsegelskyi, R. (2018). Pander: An R 'Pandoc' Writer. R package version 0.6.3. https://CRAN.R‐project.org/package=pander

[ece37881-bib-0017] De Boer, T. E. , Tas, N. , Braster, M. , Temminghoff, E. J. M. , Röling, W. F. M. , & Roelofs, D. (2012). The influence of long‐term copper contaminated agricultural soil at different pH levels on microbial communities and springtail transcriptional regulation. Environmental Science and Technology, 46(1), 60–68. 10.1021/es2013598 21882881

[ece37881-bib-0019] Deharveng, L. (1996). Soil Collembola diversity, endemism and reforestation: A case study in the Pyrenees (France). Conservation Biology, 10(1), 74–84. 10.1046/j.1523-1739.1996.10010074.x

[ece37881-bib-0020] Deharveng, L. , Bedos, A. , & Leksawasdi, P. (1989). Diversity in tropical forest soils: The Collembola of Doi Inthanon (Thailand). In Third International Seminar on Apterygota. University of Siena, Siena (pp. 317–328).

[ece37881-bib-0021] Deharveng, L. , Bedos, A. , & Lukic, M. (2020). The genus *Folsomides* in the Hòn Chông hills, Vietnam (Collembola: Isotomidae). In M. Kottelat , L. Deharveng , & P. K. L. Ng (Eds.): Antony J. Whitten (1953‐2017) memorial issue, The Raffles Bulletin of Zoology supplement (Vol. 35, pp. 32–47). Singapore: National University of Singapore ISSN 2345‐7600 (electronic) | ISSN 021‐2445 (print).

[ece37881-bib-0022] Deharveng, L. , & Suhardjono, Y. R. (1994). *Isotomiella* Bagnall, 1939 (Collembola, Isotomidae) of Sumatra (Indonesia). Tropical Zoology, 7(2), 309–323. 10.1080/03946975.1994.10539261

[ece37881-bib-0023] Devi, W. M. , Singh, T. B. , & Devi, L. J. (2011). ‘Monthly changes of collembolan population under the gradients of moisture, organic carbon and nitrogen contents in a sub‐tropical forest soil. Manipur, 2(12), 10–12.

[ece37881-bib-0024] Drescher, J. , Rembold, K. , Allen, K. , Beckschäfer, P. , Buchori, D. , Clough, Y. , Faust, H. , Fauzi, A. M. , Gunawan, D. , Hertel, D. , Irawan, B. , Jaya, I. N. S. , Klarner, B. , Kleinn, C. , Knohl, A. , Kotowska, M. M. , Krashevska, V. , Krishna, V. , Leuschner, C. , … Scheu, S. (2016). Ecological and socio‐economic functions across tropical land use systems after rainforest conversion. Philosophical Transactions of the Royal Society B: Biological Sciences, 371(1694), 20150275. 10.1098/rstb.2015.0275 PMC4843696 27114577

[ece37881-bib-0025] Edwards, D. P. , Magrach, A. , Woodcock, P. , Ji, Y. , Lim, N. T. L. , Edwards, F. A. , Larsen, T. H. , Hsu, W. W. , Benedick, S. , Khen, C. V. , Chung, A. Y. C. , Reynold, G. , Fisher, B. , Laurance, W. F. , Wilcove, D. S. , Hamer, K. C. , & Yu, D. W. (2014). Selective‐logging and oil palm: Multitaxon impacts, biodiversity indicators, and trade‐offs for conservation planning. Ecological Applications, 24(8), 2029–2049. 10.1890/14-0010.1 29185670

[ece37881-bib-0028] Fatimah, F. , Cholik, E. , & Suhardjono, Y. R. (2012). Collembola Permukaan Tanah Kebun Karet, Lampung. Bidang Zoologi Pusat Penelitian Biologi LIPI, 21(2), 17–22.

[ece37881-bib-0029] Fierer, N. , Schimel, J. P. , & Holden, P. A. (2003). Influence of drying‐rewetting frequency on soil bacterial community structure. Microbial Ecology, 45(1), 63–71. 10.1007/s00248-002-1007-2 12469245

[ece37881-bib-0030] Filser, J. (1995). The effect of green manure on the distribution of collembola in a permanent row crop. Biology and Fertility of Soils, 19(4), 303–308. 10.1007/BF00336099

[ece37881-bib-0031] Fitzherbert, E. , Struebig, M. , Morel, A. , Danielsen, F. , Bruhl, C. , Donald, P. , & Phalan, B. (2008). How will oil palm expansion affect biodiversity? Trends in Ecology and Evolution, 23(10), 538–545. 10.1016/j.tree.2008.06.012 18775582

[ece37881-bib-0032] Fox, J. , & Weisberg, S. (2019). An R companion to applied regression (3rd ed.). Sage. https://socialsciences.mcmaster.ca/jfox/Books/Companion/

[ece37881-bib-0033] Frampton, G. K. , & Van Den Brink, P. J. (2002). Influence of cropping on the species composition of epigeic Collembola in arable fields. Pedobiologia, 46(3–4), 328–337. 10.1078/0031-4056-00140

[ece37881-bib-0034] Fujii, S. , Saitoh, S. , & Takeda, H. (2014). Effects of rhizospheres on the community composition of Collembola in a temperate forest. Applied Soil Ecology, 83, 109–115. 10.1016/j.apsoil.2014.03.018

[ece37881-bib-0035] Gagic, V. , Bartomeus, I. , Jonsson, T. , Taylor, A. , Winqvist, C. , Fischer, C. , Slade, E. M. , Dewenter, I. S. , Emmerson, M. , Potts, S. G. , Tscharntke, T. , Weisser, W. , & Bommarco, R. (2015). Functional identity and diversity of animals predict ecosystem functioning better than species‐based indices. Proceedings of the Royal Society B: Biological Sciences, 282(1801), 20142620. 10.1098/rspb.2014.2620 PMC4309003 25567651

[ece37881-bib-0037] Gatto, M. , Wollni, M. , & Qaim, M. (2015). ‘Oil palm boom and land‐use dynamics in Indonesia: The role of policies and socioeconomic factors. Land Use Policy, 46, 292–303. 10.1016/j.landusepol.2015.03.001

[ece37881-bib-0038] Gilbert, N. (2012). Palm‐oil boom raises conservation concerns. Nature, 487(7405), 14–15. 10.1038/487014a 22763524

[ece37881-bib-0039] Glime, J. M. , & Wagner, D. M. (2017). Laboratory techniques: Preservation and permanent mounts. Chapt. 2‐4. Bryophyte Ecology, (3), 1–18. Michigan Technological University and the International Association of Bryologists. http://digitalcommons.mtu.edu/bryophyte‐ecology

[ece37881-bib-0041] Hawes, T. C. , & Greenslade, P. (2015). A note on scale morphology in Collembola. Zootaxa, 3925(4), 594–596. 10.11646/zootaxa.3925.4.8 25781764

[ece37881-bib-0042] Hopkin, S. P. (1997). Biology of the springtails (Insecta, Collembola). Oxford University Press.

[ece37881-bib-0043] Hothorn, T. , Bretz, F. , & Westfall, P. (2008). Simultaneous inference in general parametric models. Biometrical Journal, 50(3), 346–363. 10.1002/bimj.200810425 18481363

[ece37881-bib-0044] Jan Leaps, P. S. (2003). Multivariate analysis using CANOCO 4.5. Cambridge University Press.

[ece37881-bib-0045] Kempson, D. , Lloyd, M. , & Gheraldi, R. (1963). A new extractor for woodland litter. Pedobiologia, 3(1), 1–21.

[ece37881-bib-0046] Koh, L. P. , & Ghazoul, J. (2010). Reply to Sloan and Stork: Spatially explicit scenario analysis for reconciling agricultural expansion, forest protection, and carbon conservation in Indonesia. Proceedings of the National Academy of Sciences of the United States of America, 107(45), E172. 10.1073/pnas.1012681107 PMC2890708 20511535

[ece37881-bib-0047] Kotowska, M. M. , Leuschner, C. , Triadiati, T. , Meriem, S. , & Hertel, D. (2015). Quantifying above‐ and belowground biomass carbon loss with forest conversion in tropical lowlands of Sumatra (Indonesia). Global Change Biology, 21(10), 3620–3634. 10.1111/gcb.12979 25980371

[ece37881-bib-0048] Krashevska, V. , Klarner, B. , Widyastuti, R. , Maraun, M. , & Scheu, S. (2015). Impact of tropical lowland rainforest conversion into rubber and oil palm plantations on soil microbial communities. Biology and Fertility of Soils, 51(6), 697–705. 10.1007/s00374-015-1021-4

[ece37881-bib-0049] Krashevska, V. , Klarner, B. , Widyastuti, R. , Maraun, M. , & Scheu, S. (2016). Changes in structure and functioning of protist (testate amoebae) communities due to conversion of lowland rainforest into rubber and oil palm plantations. PLoS One, 11(7), e0160179. 10.1371/journal.pone.0160179 27463805 PMC4963170

[ece37881-bib-0050] Krashevska, V. , Kudrin, A. , Widyastuti, R. , & Scheu, S. (2019). Changes in nematode communities and functional diversity with the conversion of rainforest into rubber and oil palm plantations. Frontiers in Ecology and Evolution, 7, 1–10. 10.3389/fevo.2019.00487

[ece37881-bib-0051] Kuznetsova, N. A. (2003). Humidity and distribution of springtails. Zoologicheskii Zhurnal, 82(2), 239–247 [Ento‐mol. Rev. 83 (2): 230–238]. 10.1134/S0013873815060032

[ece37881-bib-0052] Laliberté, E. , & Legendre, P. (2010). A distance‐based framework for measuring functional diversity from multiple traits. Ecology, 91(1), 299–305. 10.1890/08-2244.1 20380219

[ece37881-bib-0053] Lawrence, K. L. , & Wise, D. H. (2000). Spider predation on forest‐floor Collembola and evidence for indirect effects on decomposition. Pedobiologia, 44, 33–39. 10.1016/j.nutres.2013.07.004

[ece37881-bib-0054] Li, Z. , Scheunemann, N. , Potapov, A. M. , Shi, L. , Pausch, J. , Scheu, S. , & Pollierer, M. M. (2020). Incorporation of root‐derived carbon into soil microarthropods varies between cropping systems. Biology and Fertility of Soils, 56, 839–851. 10.1007/s00374-020-01467-8

[ece37881-bib-0056] Martius, C. , Höfer, H. , Garcia, M. V. B. , Römbke, J. , Förster, B. , & Hanagarth, W. (2004). Microclimate in agroforestry systems in central Amazonia: Does canopy closure matter to soil organisms? Agroforestry Systems, 60, 291–304. 10.1023/B:AGFO.0000024419.20709.6c

[ece37881-bib-0057] Mateos, E. , & Greenslade, P. (2015). Towards understanding *Lepidocyrtu*s Bourlet, 1839 (Collembola, Entomobryidae) I: Diagnosis of the subgenus *Setogaster*, new records and redescriptions of species. Zootaxa, 4044(1), 105–129. 10.11646/zootaxa.4044.1.6 26624705

[ece37881-bib-0058] McFerrin, L. (2013). HDMD: Statistical analysis tools for high dimension molecular data (HDMD). R package version 1.2. https://CRAN.R‐project.org/package=HDMD

[ece37881-bib-0059] Michon, G. , & de Foresta, H. (1995). Conserving biodiversity outside protected areas: The Indonesian agro‐forest model. In Encyclopedia of biodiversity (Conserving biological diversity, 2nd ed. (90–106). UK: IUCN, Gland, Switzerland, and Cambridge. 10.1016/B978-0-12-384719-5.00359-2

[ece37881-bib-0061] Muturi, J. J. , Mbugi, J. P. , Mueke, J. M. , Lagerlöf, J. , Mungatu, J. K. , Nyamasyo, G. , & Gikungu, M. (2011). Effect of integrated soil fertility management interventions on the abundance and diversity of soil Collembola in Embu and Taita Districts, Kenya. Tropical and Subtropical Agroecosystems, 13(1), 37–42.

[ece37881-bib-0062] Nazarreta, R. , Hartke, T. R. , Hidayat, P. , Scheu, S. , Buchori, D. , & Drescher, J. (2020). Rainforest conversion to smallholder plantations of rubber or oil palm leads to species loss and community shifts in canopy ants (Hymenoptera: Formicidae). Myrmecological News, 30, 175–186. 10.25849/myrmecol.news_030:175

[ece37881-bib-0063] Nordhausen, K. , Sirkia, S. , Oja, H. , & Tyler, D. E. (2018). ICSNP: Tools for multivariate nonparametrics. R package version 1.1‐1. https://CRAN.R‐project.org/package=ICSNP

[ece37881-bib-0064] Oksanen, J. , Blanchet, F. G. , Friendly, M. , Kindt, R. , Legendre, P. , McGlinn, D. , Minchin, P. R. , O'Hara, R. B. , Simpson, G. L. , Solymos, P. , Henry, M. , Stevens, H. , Szoecs, E. , & Wagner, H. (2019). vegan: Community ecology package. R package version 2.5‐6. https://CRAN.R‐project.org/package=vegan

[ece37881-bib-0065] Pey, B. , Nahmani, J. , Auclerc, A. , Capowiez, Y. , Cluzeau, D. , Cortet, J. , Decaëns, T. , Deharveng, L. , Dubs, F. , Joimel, S. , Briard, C. , Grumiaux, F. , Laporte, M.‐A. , Pasquet, A. , Pelosi, C. , Pernin, C. , Ponge, J.‐F. , Salmon, S. , Santorufo, L. , & Hedde, M. (2014). Current use of and future needs for soil invertebrate functional traits in community ecology. Basic and Applied Ecology, 15(3), 194–206. 10.1016/j.baae.2014.03.007

[ece37881-bib-0068] Ponge, J. F. (1993). Biocenoses of Collembola in Atlantic temperate grass‐woodland ecosystems. Pedobiologia, 37(4), 223–244. ⟨hal‐00506012⟩.

[ece37881-bib-0070] Ponge, J. F. , & Salmon, S. (2013). Spatial and taxonomic correlates of species and species trait assemblages in soil invertebrate communities. Pedobiologia, 56(3), 129–136. 10.1016/j.pedobi.2013.02.001

[ece37881-bib-0071] Potapov, A. M. , Dupérré, N. , Jochum, M. , Dreczko, K. , Klarner, B. , Barnes, A. D. , Krashevska, V. , Rembold, K. , Kreft, H. , Brose, U. , Widyastuti, R. , Harms, D. , & Scheu, S. (2020). Functional losses in ground spider communities due to habitat structure degradation under tropical land‐use change. Ecology, 101(3), e02957. 10.1002/ecy.2957 31840252

[ece37881-bib-0072] Potapov, A. M. , Goncharov, A. A. , Tsurikov, S. M. , Tully, T. , & Tiunov, A. V. (2016). Assimilation of plant‐derived freshly fixed carbon by soil collembolans: Not only via roots? Pedobiologia, 59(4), 189–193. 10.1016/j.pedobi.2016.07.002

[ece37881-bib-0073] Potapov, A. M. , Klarner, B. , Sandmann, D. , Widyastuti, R. , & Scheu, S. (2019). Linking size spectrum, energy flux and trophic multifunctionality in soil food webs of tropical land‐use systems. Journal of Animal Ecology, 88(12), 1845–1859. 10.1111/1365-2656.13027 31111468

[ece37881-bib-0074] Potapov, M. (2012). Taxonomy of the *Proisotoma* complex. Transactions of the American Microscopical Society, 49(1), 38–49. 10.25674/so-91-1-01

[ece37881-bib-0075] Potapov, M. B. , & Starostenko, E. V. (2002). Taxonomical notes on the species of the genus *Isotomurus* (Collembola: Isotomidae) with the “balteatus”‐like colouration. Russian Entomological Journal, 11(4), 331–333.

[ece37881-bib-0076] R Core Team (2017). R: A language and environment for statistical computing. R Foundation for Statistical Computing. https://www.r‐project.org/

[ece37881-bib-0077] Raymond‐Léonard, L. J. , Gravel, D. , & Handa, I. T. (2019). A novel set of traits to describe Collembola mouthparts: Taking a bite out of the broad chewing mandible classification. Soil Biology and Biochemistry, 138, 107608. 10.1016/j.soilbio.2019.107608

[ece37881-bib-0104] Rembold, K. , Mangopo, H. , Tjitrosoedirdjo, S. S. , & Kreft, H. (2017). Plant diversity, forest dependency, and alien plant invasions in tropical agricultural landscapes. Biological Conservation, 213(A), 234–242. https://www.sciencedirect.com/science/article/abs/pii/S0006320717303968

[ece37881-bib-0078] Rojas, A. B. , Castano‐Meneses, G. , Palacioos‐Vargas, J. G. , & Garcia‐Calderon, N. E. (2009). Oribatid mites and springtails from a coffee plantation in Sierra Sur, Oaxaca, Mexico. Pesquisa Agropecuária Brasileira, 44(8), 988–995. 10.1590/s0100-204x2009000800030

[ece37881-bib-0103] Rousk, J. , Baath, E. , Brookes, P. C. , Lauber, Christian L , Lozupone, C. , Caporaso, J. G. , Knight, R. , & Fierer, N. (2010). Soil bacterial and fungal communities across a pH gradient in an arable soil. The ISME Journal, 4(10), 1340–1351. https://www.nature.com/articles/ismej201058 20445636 10.1038/ismej.2010.58

[ece37881-bib-0079] Rusek, J. (1998). Biodiversity of Collembola and their functional role in the ecosystem. Biodiversity and Conservation, 7(9), 1207–1219. 10.1023/A:1008887817883

[ece37881-bib-0080] Salmon, S. , & Ponge, J. F. (2012). Species traits and habitats in springtail communities: A regional scale study. Pedobiologia, 55(6), 295–301. 10.1016/j.pedobi.2012.05.003

[ece37881-bib-0081] Salmon, S. , Ponge, J. F. , Gachet, S. , Deharveng, L. , Lefebvre, N. , & Delabrosse, F. (2014). Linking species, traits and habitat characteristics of Collembola at European scale. Soil Biology and Biochemistry, 75, 73–85. 10.1016/j.soilbio.2014.04.002

[ece37881-bib-0105] Salmon, S. , Ponge, J. F. , Gachet, S. , Deharveng, L. , Levebvre, N. , & Delabrosse, F. (2014). Linking species, traits and habitat characteristics of Collembola at European scale. Soil Biology and Biochemistry, 75, 73–85. https://www.sciencedirect.com/science/article/abs/pii/S0038071714001229

[ece37881-bib-0082] Schulz, G. , Schneider, D. , Brinkmann, N. , Edy, N. , Daniel, R. , Polle, A. , Scheu, S. , & Krashevska, V. (2019). Changes in trophic groups of protists with conversion of rainforest into rubber and oil palm plantations. Frontiers in Microbiology, 10(240), 1–14. 10.3389/fmicb.2019.00240 30809219 PMC6380168

[ece37881-bib-0084] Selvany, R. (2018). Kelimpahan dan keanekaragaman collembola pada lima tipe penggunaan lahan di Kapuas Hulu Kalimantan Barat. Thesis, IPB (ID).

[ece37881-bib-0085] Son, J. , Shin, K. , & Cho, K. (2009). Response surface model for predicting chronic toxicity of cadmium to Paronychiurus kimi (Collembola), with a special emphasis on the importance of soil characteristics in the reproduction test. Chemosphere, 77(7), 889–894. 10.1016/j.chemosphere.2009.08.047 19783280

[ece37881-bib-0086] Sousa, J. P. , Bolger, T. , da Gama, M. M. , Lukkari, T. , Ponge, J. F. , Simon, C. , Traser, G. , Vanbergen, A. J. , Brennan, A. , Dubs, F. , Ivitis, E. , Keating, A. , Stofer, S. , & Watt, A. (2006). Changes in Collembola richness and diversity along a gradient of land‐use intensity: A pan European study. Pedobiologia, 50(2), 147–156. 10.1016/j.pedobi.2005.10.005

[ece37881-bib-0087] Suhardjono, Y. R. , Deharveng, L. , & Bedos, A. (2012). Collembola (ekor pegas): Biologi, klasifikasi, ekologi. PT Vega Briantama Vandanesia.

[ece37881-bib-0088] Susanti, W. I. , Pollierer, M. M. , Widyastuti, R. , Scheu, S. , & Potapov, A. (2019). Conversion of rainforest to oil palm and rubber plantations alters energy channels in soil food webs. Ecology and Evolution, 9(16), 9027–9039. 10.1002/ece3.5449 31463001 PMC6706186

[ece37881-bib-0089] Tao, H. H. , Snaddon, J. L. , Slade, E. M. , Henneron, L. , Caliman, J. P. , & Willis, K. J. (2018). Application of oil palm empty fruit bunch effects on soil biota and functions: A case study in Sumatra. Indonesia. Agriculture, Ecosystem & Environment, 256, 105–113. 10.1016/j.agee.2017.12.012

[ece37881-bib-0090] ter Braak, C. J. , & Smilauer, P. (2012). Canoco reference manual and user's guide: Software for ordination, version 5.0.

[ece37881-bib-0091] Titeux, N. , Henle, K. , Mihoub, J. B. , Regos, A. , Geijzendorffer, I. R. , Cramer, W. , Verburg, P. H. , & Brotons, L. (2016). Biodiversity scenarios neglect future land‐use changes. Global Change Biology, 22(7), 2505–2515. 10.1111/gcb.13272 26950650

[ece37881-bib-0092] Van Straalen, N. M. , Timmermans, M. J. T. N. , Roelofs, D. , & Berg, M. P. (2008). Apterygota in the spotlights of ecology, evolution and genomics. European Journal of Soil Biology, 44(5–6), 452–457. 10.1016/j.ejsobi.2008.07.003

[ece37881-bib-0093] Vandewalle, M. , de Bello, F. , Berg, M. P. , Bolger, T. , Doledec, S. , Dubs, F. , Feld, C. K. , Harrington, R. , Harrison, P. A. , Lavorel, S. , da Silva, P. M. , Moretti, M. , Niemelä, J. , Santos, P. , Sattler, T. , Sousa, J. P. , Sykes, M. T. , Vanbergen, A. J. , & Woodcock, B. A. (2010). Functional traits as indicators of biodiversity response to land use changes across ecosystems and organisms. Biodiversity and Conservation, 19(10), 2921–2947. 10.1007/s10531-010-9798-9

[ece37881-bib-0094] Venables, W. N. , & Ripley, B. D. (2002). Modern applied statistics with S (4th ed.). Springer. ISBN 0‐387‐95457‐0.

[ece37881-bib-0095] Violle, C. , Navas, M. L. , Vile, D. , Kazakou, E. , Fortunel, C. , Hummel, I. , & Garnier, E. (2007). Let the concept of trait be functional!. Oikos, 116(5), 1–11. 10.1111/j.2007.0030-1299.15559.x

[ece37881-bib-0096] Warino, J. , Widyastuti, R. , Suhardjono, Y. R. , & Nugroho, B. (2017). Keanekaragaman dan kelimpahan Collembola pada perkebunan kelapa sawit di Kecamatan Bajubang, Jambi. Journal Entomologi Indonesia, 14(2), 51–57. 10.5994/jei.14.2.51

[ece37881-bib-0099] Widrializa, W. , Widyastuti, R. , Santosa, D. A. , & Djajakirana, G. (2015). The diversity and abundance of springtail (Collembola) on forests and smallholder in Jambi. Jurnal Tanah Tropika, 20(3), 173–180. 10.5400/jts.2015.20.3

[ece37881-bib-0100] Winck, B. R. , de Sá, E. L. S. , Rigotti, V. M. , & Chauvat, M. (2017). Relationship between land‐use types and functional diversity of epigeic Collembola in Southern Brazil. Applied Soil Ecology, 109, 49–59. 10.1016/j.apsoil.2016.09.021

[ece37881-bib-0101] Zhang, X. F. , Zhao, L. , Xu, S. J. Jr , Liu, Y. Z. , Liu, H. S. Y. , & Cheng, G. D. (2013). Soil moisture effect on bacterial and fungal community in Beilu River (Tibetan Plateau) permafrost soils with different vegetation types. Journal of Applied Microbiology, 114(4), 1054–1065. 10.1111/jam.12106 23241008

[ece37881-bib-0102] Zuur, A. , Ieno, E. N. , Walker, N. , Saveliev, A. A. , & Smith, G. M. (2009). Mixed effects models and extensions in ecology with R. Springer Science & Business Media.

