# Supplementary material for: Conversion of rainforest into oil palm and rubber plantations affects the functional composition of litter and soil Collembola
Source: Ecol Evol. 2021 Jul 13;11(15):10686–708. doi: 10.1002/ece3.7881 (PMC8328430; doi:10.1002/ece3.7881)
Supplement: Supplementary file 1 — Supplementary Material [file ECE3-11-10686-s001.docx]

**Supplementary Material**

**Supplementary Table S1. Full list of species and traits**

| **No.** | **Species** | **Pigmentation** | **Ocelli** | **Antennae** | **Furca** | **Abdomen** | **Scales** | **PAO** | **Mouthparts** | **Empodial appendage** | **Size Class** |
| --- | --- | --- | --- | --- | --- | --- | --- | --- | --- | --- | --- |
| 1 | *Acrocyrtus* sp.1 | Absent | >=6+6 | Normal, 4 segments | Whip-shaped | Abdomen IV elongated | Scales smooth | PAO absent | Chewing | Present | Medium size |
| 2 | *Acrocyrtus* sp.2 | Diffuse | >=6+6 | Normal, 4 segments | Whip-shaped | Abdomen IV elongated | Scales smooth | PAO absent | Chewing | Present | Large size |
| 3 | *Acrocyrtus* sp.3 | Pattern | >=6+6 | Normal, 4 segments | Whip-shaped | Abdomen IV elongated | Scales smooth | PAO absent | Chewing | Present | Large size |
| 4 | *Allacma* sp.1 | Pattern | >=6+6 | Antennae III and/or IV subdivided | Straight | Spherical body | Absent | PAO absent | Chewing | Present | Small size |
| 5 | *Dahlcyrtus* sp. 1 | Pattern | >=6+6 | Normal, 4 segments | Whip-shaped | Abdomen IV elongated | Scales smooth | PAO absent | Chewing | Present | Large size |
| 6 | *Ascocyrtus cinctus* | Pattern | >=6+6 | Normal, 4 segments | Whip-shaped | Abdomen IV elongated | Scales smooth | PAO absent | Chewing | Present | Medium size |
| 7 | *Ascocyrtus* sp.1 | Pattern | >=6+6 | Normal, 4 segments | Whip-shaped | Abdomen IV elongated | Scales smooth | PAO absent | Chewing | Present | Medium size |
| 8 | *Callyntrura* sp.1 | Pattern | >=6+6 | Antennae I very long | Long cylindric | Abdomen IV elongated | Scales smooth | PAO absent | Chewing | Present | Large size |
| 9 | *Callyntrura* sp.2 | Pattern | >=6+6 | Antennae I very long | Long cylindric | Abdomen IV elongated | Scales smooth | PAO absent | Chewing | Present | Medium size |
| 10 | *Callyntrura* sp.3 | Pattern | >=6+6 | Antennae I very long | Long cylindric | Abdomen IV elongated | Scales smooth | PAO absent | Chewing | Present | Medium size |
| 11 | *Callyntrura* sp.4 | Pattern | >=6+6 | Antennae I very long | Long cylindric | Abdomen IV elongated | Scales smooth | PAO absent | Chewing | Present | Large size |
| 12 | *Coecobrya* sp.1 | Absent | Absent | Normal, 4 segments | Whip-shaped | Abdomen IV elongated | Absent | PAO absent | Chewing | Present | Medium size |
| 13 | *Cyphoderopsis* sp.1 | Absent | Absent | Normal, 4 segments | Long cylindric | Abdomen IV elongated | Scales smooth | PAO absent | Chewing | Present | Medium size |
| 14 | *Cyphoderus* sp.1 | Absent | Absent | Normal, 4 segments | Straight | Abdomen IV elongated | Scales smooth | PAO absent | Chewing | Present | Medium size |
| 15 | *Dicranocentrus* sp.1 | Pattern | >=6+6 | Antennae I and/or II subdivided | Whip-shaped | Not modified | Scales smooth | PAO absent | Chewing | Present | Large size |
| 16 | *Dicranocentrus* sp.2 | Pattern | >=6+6 | Antennae I and/or II subdivided | Whip-shaped | Not modified | Scales smooth | PAO absent | Chewing | Present | Medium size |
| 17 | *Dicranocentrus* sp.3 | Pattern | >=6+6 | Antennae I and/or II subdivided | Whip-shaped | Not modified | Scales smooth | PAO absent | Chewing | Present | Medium size |
| 18 | *Entomobrya* sp.1 | Diffuse | >=6+6 | Normal, 4 segments | Whip-shaped | Abdomen IV elongated | Absent | PAO absent | Chewing | Present | Small size |
| 19 | *Folsomides centralis* | Diffuse | >=6+6 | Normal, 4 segments | Straight | Not modified | Absent | PAO simple | Chewing | Present | Small size |
| 20 | *Folsomides parvulus* | Absent | 2+2 | Normal, 4 segments | Straight | Not modified | Absent | PAO simple | Chewing | Present | Small size |
| 21 | *Folsomina infelicia* | Absent | Absent | Normal, 4 segments | Straight | Abdomen IV to VI or V to VI fused | Absent | PAO absent | Chewing | Present | Small size |
| 22 | *Folsomina onychiurina* | Absent | Absent | Normal, 4 segments | Straight | Abdomen IV to VI or V to VI fused | Absent | PAO absent | Chewing | Present | Medium size |
| 23 | *Alloscopus tetracanthus* | Absent | Absent | Antennae I and/or II subdivided | Whip-shaped | Not modified | Scales smooth | PAO simple | Chewing | Present | Large size |
| 24 | *Homidia cingula* | Pattern | >=6+6 | Normal, 4 segments | Whip-shaped | Abdomen IV elongated | Absent | PAO absent | Chewing | Present | Large size |
| 25 | *Isotomiella cf. alulu* | Absent | Absent | Normal, 4 segments | Whip-shaped | Abdomen IV to VI or V to VI fused | Absent | PAO absent | Chewing | Present | Small size |
| 26 | *Isotomiella cf. minor* | Absent | Absent | Normal, 4 segments | Whip-shaped | Abdomen IV to VI or V to VI fused | Absent | PAO absent | Chewing | Present | Small size |
| 27 | *Isotomiella symetrimucronata* | Absent | Absent | Normal, 4 segments | Whip-shaped | Abdomen IV to VI or V to VI  fused | Absent | PAO absent | Chewing | Present | Small size |
| 28 | *Isotomodes* sp.1 | Absent | Absent | Normal, 4 segments | Straight | Not modified | Absent | PAO simple | Chewing | Present | Small size |
| 29 | *Isotomurus cf. parabalteatus* | Pattern | >=6+6 | Normal, 4 segments | Whip-shaped | Not modified | Absent | PAO simple | Chewing | Present | Medium size |
| 30 | *Lanocyrtus* sp.1 | Pattern | >=6+6 | Normal, 4 segments | Whip-shaped | Abdomen IV elongated | Scales smooth | PAO absent | Chewing | Present | Small size |
| 31 | *Lanocyrtus* sp.2 | Pattern | >=6+6 | Normal, 4 segments | Whip-shaped | Abdomen IV elongated | Scales smooth | PAO absent | Chewing | Present | Medium size |
| 32 | *Lepidocyrtus* sp.1 | Diffuse | >=6+6 | Normal, 4 segments | Whip-shaped | Abdomen IV elongated | Scales smooth | PAO absent | Chewing | Present | Medium size |
| 33 | *Lepidocyrtus* sp.2 | Diffuse | >=6+6 | Normal, 4 segments | Whip-shaped | Abdomen IV elongated | Scales smooth | PAO absent | Chewing | Present | Medium size |
| 34 | *Lepidonella* sp.1 | Pattern | >=6+6 | Normal, 4 segments | Long cylindric | Abdomen IV elongated | Scales smooth | PAO absent | Chewing | Present | Medium size |
| 35 | *Megalothorax cf. minimus* | Absent | Absent | Normal, 4 segments | Straight | Spherical body | Absent | PAO absent | Chewing | Present | Small size |
| 36 | *Onychiuridae gen.* sp. | Absent | Absent | Normal, 4 segments | Absent | Not modified | Absent | PAO complex | Chewing | Present | Small size |
| 37 | *Pararrhopalites* sp.1 | Pattern | >=6+6 | Antennae III and/or IV subdivided | Straight | Spherical body | Absent | PAO absent | Chewing | Present | Small size |
| 38 | *Pseudachorutella cf. stachi* | Intensive | >=6+6 | Normal, 4 segments | Straight | Not modified | Absent | PAO absent | piercing/sucking | Absent | Medium size |
| 39 | *Pseudachorutes* sp.1 | Intensive | >=6+6 | Normal, 4 segments | Straight | Not modified | Absent | PAO complex | piercing/sucking | Absent | Medium size |
| 40 | *Pseudachorutes* sp.2 | Intensive | >=6+6 | Normal, 4 segments | Straight | Not modified | Absent | PAO complex | piercing/sucking | Absent | Small size |
| 41 | *Pseudosinella* sp.1 | Absent | Absent | Normal, 4 segments | Whip-shaped | Abdomen IV elongated | Scales smooth | PAO absent | Chewing | Present | Medium size |
| 42 | *Ptenothrix* sp.1 | Pattern | >=6+6 | Antennae IV shorter than III | Straight | Spherical body | Absent | PAO absent | Chewing | Present | Medium size |
| 43 | *Rambutsinella* sp. (cf. scopae) | Diffuse | 2 + 2 | Normal, 4 segments | Whip-shaped | Abdomen IV elongated | Scales smooth | PAO absent | Chewing | Present | Medium size |
| 44 | Salina sp.1 | Pattern | >=6+6 | Normal, 4 segments | Long cylindric | Abdomen IV elongated | Absent | PAO absent | Chewing | Present | Large size |
| 45 | *Sminthuridae gen*. sp.1 | Diffuse | >=6+6 | Normal, 4 segments | Long cylindric | Spherical body | Absent | PAO absent | Chewing | Present | Small size |
| 46 | *Sminthurides* sp.1 | Diffuse | >=6+6 | Normal, 4 segments | Straight | Spherical body | Absent | PAO absent | Chewing | Present | Small size |
| 47 | *Sphaeridia* sp.1 | Intensive | >=6+6 | Normal, 4 segments | Straight | Spherical body | Absent | PAO absent | Chewing | Present | Small size |
| 48 | *Sphaeridia* sp.2 | Intensive | >=6+6 | Normal, 4 segments | Straight | Spherical body | Absent | PAO absent | Chewing | Present | Small size |
| 49 | *Sphyroteca* sp.1 | Pattern | >=6+6 | Antennae III and/or IV subdivided | Straight | Spherical body | Absent | PAO absent | Chewing | Present | Small size |
| 50 | *Superodontella* sp.1 | Intensive | 5 + 5 | Normal, 4 segments | Straight | Not modified | Absent | PAO complex | Not Chewing | Absent | Small size |
| 51 | *Telobella* sp.1 | Intensive | 2 + 2 | Normal, 4 segments | Absent | Not modified | Absent | PAO absent | piercing/sucking | Absent | Large size |
| 52 | *Thalassaphorura* sp.1 | Absent | Absent | Normal, 4 segments | Absent | Not modified | Absent | PAO complex | Chewing | Absent | Small size |
| 53 | *Tullbergiinae gen.* sp. | Absent | Absent | Normal, 4 segments | Absent | Not modified | Absent | PAO complex | Chewing | Absent | Medium size |
| 54 | *Xenylla* sp.1 | Diffuse | 4 + 4 | Normal, 4 segments | Straight | Not modified | Absent | PAO absent | Chewing | Absent | Small size |

**Supplementary Table S2. Species matrix of Collembola (individuals per plot) in litter layer**

| Systems | Plot | Year | *Acr1* | *Acr2* | *Allo* | *Call1* | *Asco* | *Dicr1* | *Folce* | *Folspa* | *Homci* | *Isomi* | *Lep1* | *Mega* | *Ony* | *Par1* | *Pseu1* | *Ram* | *Spha1* | *Spha2* | *Sphy1* | *Sup1* | *Xen1* |
| --- | --- | --- | --- | --- | --- | --- | --- | --- | --- | --- | --- | --- | --- | --- | --- | --- | --- | --- | --- | --- | --- | --- | --- |
| Rainforest | BF1b | 2013 | 1 | 12 | 0 | 2 | 3 | 1 | 0 | 19 | 0 | 21 | 0 | 0 | 2 | 4 | 1 | 4 | 0 | 0 | 2 | 0 | 0 |
| Rainforest | BF2b | 2013 | 0 | 0 | 0 | 0 | 0 | 0 | 21 | 0 | 0 | 2 | 0 | 0 | 30 | 0 | 0 | 0 | 1 | 0 | 0 | 0 | 0 |
| Rainforest | BF3b | 2013 | 0 | 10 | 0 | 1 | 0 | 4 | 3 | 0 | 9 | 0 | 0 | 1 | 13 | 4 | 10 | 7 | 3 | 0 | 0 | 2 | 3 |
| Rainforest | BF4b | 2013 | 0 | 0 | 0 | 0 | 5 | 4 | 0 | 1 | 0 | 28 | 6 | 0 | 16 | 6 | 0 | 0 | 1 | 0 | 0 | 0 | 0 |
| Rainforest | HF1b | 2013 | 0 | 0 | 6 | 0 | 0 | 6 | 9 | 0 | 0 | 15 | 18 | 0 | 0 | 16 | 14 | 0 | 0 | 11 | 0 | 0 | 5 |
| Rainforest | HF2b | 2013 | 0 | 0 | 0 | 0 | 0 | 0 | 0 | 0 | 0 | 0 | 0 | 0 | 1 | 0 | 0 | 0 | 0 | 0 | 0 | 0 | 0 |
| Rainforest | HF3b | 2013 | 0 | 0 | 0 | 0 | 0 | 0 | 0 | 0 | 0 | 3 | 8 | 1 | 2 | 21 | 18 | 0 | 0 | 3 | 0 | 0 | 0 |
| Rainforest | HF4b | 2013 | 0 | 1 | 0 | 0 | 0 | 4 | 0 | 0 | 0 | 6 | 45 | 0 | 0 | 18 | 1 | 4 | 0 | 2 | 0 | 0 | 0 |
| Rainforest | BF1b | 2016 | 0 | 0 | 8 | 0 | 12 | 0 | 0 | 4 | 0 | 16 | 0 | 0 | 0 | 0 | 64 | 8 | 0 | 0 | 0 | 4 | 0 |
| Rainforest | BF2b | 2016 | 4 | 0 | 0 | 8 | 0 | 0 | 12 | 32 | 0 | 36 | 0 | 0 | 0 | 0 | 4 | 4 | 0 | 12 | 0 | 0 | 0 |
| Rainforest | BF3b | 2016 | 0 | 0 | 0 | 0 | 0 | 0 | 16 | 0 | 0 | 0 | 0 | 0 | 4 | 0 | 0 | 0 | 0 | 0 | 0 | 0 | 0 |
| Rainforest | HF1b | 2016 | 0 | 0 | 0 | 0 | 0 | 0 | 0 | 0 | 0 | 12 | 0 | 0 | 0 | 0 | 0 | 0 | 0 | 0 | 0 | 0 | 0 |
| Rainforest | HF2b | 2016 | 0 | 0 | 0 | 0 | 12 | 0 | 0 | 0 | 0 | 8 | 0 | 0 | 0 | 0 | 16 | 0 | 0 | 0 | 4 | 12 | 16 |
| Rainforest | HF3b | 2016 | 0 | 0 | 4 | 0 | 36 | 0 | 28 | 8 | 0 | 68 | 0 | 0 | 0 | 0 | 16 | 40 | 0 | 0 | 0 | 0 | 4 |
| Rainforest | HFr1b | 2016 | 0 | 0 | 0 | 0 | 8 | 0 | 0 | 0 | 0 | 4 | 0 | 0 | 0 | 4 | 0 | 0 | 0 | 0 | 0 | 0 | 0 |
| Rainforest | HFr2b | 2016 | 0 | 0 | 8 | 0 | 16 | 0 | 16 | 0 | 0 | 84 | 0 | 0 | 0 | 4 | 0 | 0 | 0 | 0 | 12 | 0 | 16 |
| Rainforest | HFr3b | 2016 | 0 | 0 | 0 | 0 | 4 | 0 | 4 | 0 | 0 | 4 | 0 | 0 | 0 | 0 | 0 | 0 | 0 | 0 | 0 | 0 | 8 |
| Rainforest | HFr4b | 2016 | 0 | 0 | 0 | 0 | 4 | 0 | 0 | 0 | 0 | 0 | 0 | 0 | 0 | 0 | 4 | 0 | 0 | 0 | 4 | 4 | 0 |
| Jungle rubber | BJ2b | 2013 | 0 | 2 | 0 | 0 | 14 | 0 | 21 | 2 | 0 | 2 | 0 | 1 | 0 | 0 | 0 | 2 | 14 | 10 | 0 | 0 | 0 |
| Jungle rubber | BJ3b | 2013 | 0 | 0 | 1 | 0 | 0 | 0 | 98 | 7 | 10 | 7 | 0 | 0 | 0 | 0 | 0 | 0 | 0 | 13 | 0 | 0 | 0 |
| Jungle rubber | BJ4b | 2013 | 0 | 0 | 0 | 0 | 0 | 0 | 21 | 16 | 0 | 4 | 0 | 0 | 0 | 0 | 0 | 0 | 0 | 0 | 0 | 0 | 0 |
| Jungle rubber | BJ5b | 2013 | 0 | 0 | 0 | 0 | 0 | 0 | 12 | 11 | 0 | 0 | 0 | 0 | 0 | 0 | 0 | 0 | 0 | 0 | 0 | 0 | 1 |
| Jungle rubber | HJ1b | 2013 | 0 | 4 | 0 | 1 | 1 | 0 | 68 | 81 | 0 | 0 | 0 | 0 | 0 | 0 | 0 | 0 | 0 | 0 | 2 | 0 | 0 |
| Jungle rubber | HJ2b | 2013 | 0 | 1 | 0 | 0 | 5 | 0 | 15 | 2 | 18 | 32 | 0 | 0 | 0 | 0 | 14 | 0 | 0 | 0 | 0 | 0 | 0 |
| Jungle rubber | HJ3b | 2013 | 0 | 0 | 0 | 0 | 1 | 0 | 1 | 0 | 0 | 0 | 0 | 0 | 0 | 1 | 1 | 0 | 0 | 0 | 1 | 0 | 0 |
| Jungle rubber | HJ4b | 2013 | 0 | 0 | 0 | 0 | 1 | 0 | 13 | 17 | 0 | 10 | 0 | 0 | 0 | 0 | 0 | 0 | 0 | 3 | 4 | 0 | 0 |
| Oil palm | BO2b | 2013 | 0 | 0 | 0 | 0 | 0 | 0 | 0 | 0 | 0 | 0 | 2 | 0 | 4 | 0 | 0 | 0 | 1 | 0 | 0 | 0 | 0 |
| Oil palm | BO3b | 2013 | 0 | 0 | 4 | 0 | 0 | 0 | 32 | 0 | 0 | 0 | 3 | 2 | 5 | 5 | 8 | 0 | 0 | 0 | 0 | 0 | 1 |
| Systems | Plot | Year | *Acr1* | *Acr2* | *Allo* | *Call1* | *Asco* | *Dicr1* | *Folce* | *Folspa* | *Homci* | *Isomi* | *Lep1* | *Mega* | *Ony* | *Par1* | *Pseu1* | *Ram* | *Spha1* | *Spha2* | *Sphy1* | *Sup1* | *Xen1* |
| Oil palm | BO4b | 2013 | 3 | 0 | 0 | 0 | 0 | 0 | 0 | 0 | 0 | 6 | 3 | 0 | 0 | 0 | 0 | 0 | 0 | 1 | 0 | 0 | 0 |
| Oil palm | BO5b | 2013 | 0 | 0 | 0 | 0 | 0 | 0 | 12 | 3 | 0 | 3 | 0 | 0 | 3 | 0 | 5 | 0 | 0 | 0 | 0 | 0 | 0 |
| Oil palm | HO1b | 2013 | 0 | 2 | 0 | 0 | 0 | 0 | 25 | 0 | 0 | 0 | 0 | 0 | 0 | 0 | 3 | 0 | 0 | 0 | 0 | 0 | 0 |
| Oil palm | HO2b | 2013 | 0 | 0 | 0 | 0 | 0 | 0 | 0 | 0 | 0 | 10 | 1 | 0 | 0 | 0 | 0 | 0 | 0 | 0 | 0 | 0 | 0 |
| Oil palm | HO3b | 2013 | 0 | 0 | 0 | 0 | 0 | 0 | 0 | 0 | 0 | 13 | 1 | 0 | 1 | 0 | 0 | 0 | 0 | 0 | 0 | 0 | 1 |
| Oil palm | BO1b | 2016 | 8 | 0 | 4 | 0 | 0 | 4 | 12 | 8 | 0 | 52 | 0 | 0 | 0 | 0 | 0 | 192 | 0 | 8 | 0 | 0 | 0 |
| Oil palm | BO2b | 2016 | 0 | 0 | 0 | 0 | 0 | 0 | 4 | 0 | 0 | 0 | 0 | 0 | 0 | 0 | 0 | 4 | 0 | 0 | 0 | 0 | 0 |
| Oil palm | BO3b | 2016 | 4 | 0 | 0 | 0 | 0 | 0 | 12 | 12 | 0 | 0 | 0 | 0 | 0 | 0 | 0 | 0 | 0 | 0 | 0 | 0 | 0 |
| Oil palm | BO4b | 2016 | 0 | 0 | 0 | 0 | 0 | 0 | 48 | 0 | 0 | 0 | 0 | 0 | 0 | 0 | 4 | 0 | 0 | 0 | 0 | 0 | 0 |
| Oil palm | HO1b | 2016 | 0 | 0 | 0 | 0 | 0 | 0 | 0 | 0 | 0 | 0 | 0 | 0 | 0 | 0 | 0 | 0 | 0 | 0 | 0 | 0 | 0 |
| Oil palm | HO2b | 2016 | 0 | 0 | 0 | 0 | 0 | 0 | 16 | 0 | 0 | 0 | 0 | 0 | 8 | 0 | 0 | 0 | 0 | 0 | 0 | 0 | 0 |
| Oil palm | HO3b | 2016 | 0 | 0 | 0 | 0 | 0 | 0 | 4 | 0 | 0 | 0 | 0 | 0 | 0 | 0 | 0 | 0 | 0 | 0 | 0 | 0 | 0 |
| Oil palm | HO4b | 2016 | 0 | 0 | 0 | 0 | 0 | 0 | 4 | 0 | 0 | 0 | 0 | 0 | 0 | 0 | 0 | 8 | 0 | 0 | 0 | 0 | 0 |
| Oil palm | HO4rb | 2016 | 0 | 0 | 0 | 0 | 0 | 0 | 0 | 0 | 0 | 20 | 0 | 0 | 0 | 0 | 0 | 0 | 0 | 0 | 0 | 0 | 0 |
| Oil palm | HOr2b | 2016 | 0 | 0 | 0 | 0 | 0 | 0 | 12 | 0 | 0 | 0 | 0 | 0 | 0 | 0 | 0 | 0 | 0 | 0 | 0 | 0 | 0 |
| Oil palm | HOr3b | 2016 | 0 | 0 | 0 | 0 | 0 | 0 | 20 | 4 | 0 | 0 | 0 | 0 | 0 | 0 | 0 | 0 | 0 | 0 | 0 | 0 | 0 |
| Rubber | BR1b | 2013 | 0 | 0 | 0 | 0 | 16 | 0 | 12 | 2 | 1 | 8 | 0 | 5 | 0 | 0 | 6 | 5 | 0 | 0 | 0 | 0 | 0 |
| Rubber | BR2b | 2013 | 3 | 0 | 0 | 0 | 6 | 0 | 7 | 0 | 0 | 0 | 0 | 0 | 0 | 0 | 0 | 0 | 0 | 0 | 3 | 0 | 0 |
| Rubber | BR3b | 2013 | 0 | 0 | 0 | 0 | 18 | 0 | 9 | 8 | 13 | 5 | 3 | 0 | 0 | 2 | 5 | 11 | 0 | 0 | 0 | 0 | 0 |
| Rubber | HR1b | 2013 | 0 | 0 | 0 | 0 | 25 | 0 | 46 | 0 | 1 | 18 | 0 | 4 | 0 | 0 | 4 | 4 | 0 | 0 | 0 | 0 | 0 |
| Rubber | HR2b | 2013 | 2 | 0 | 0 | 0 | 14 | 0 | 20 | 0 | 13 | 0 | 0 | 7 | 0 | 1 | 0 | 0 | 0 | 4 | 0 | 0 | 0 |
| Rubber | HR3b | 2013 | 0 | 0 | 1 | 0 | 0 | 0 | 8 | 1 | 1 | 0 | 0 | 0 | 0 | 0 | 3 | 0 | 0 | 0 | 0 | 0 | 0 |
| Rubber | HR4b | 2013 | 0 | 0 | 0 | 0 | 2 | 0 | 2 | 4 | 0 | 0 | 0 | 0 | 0 | 0 | 1 | 0 | 0 | 0 | 0 | 0 | 0 |
| Rubber | BR1b | 2016 | 0 | 0 | 0 | 0 | 8 | 0 | 4 | 0 | 0 | 0 | 0 | 0 | 0 | 0 | 0 | 0 | 0 | 0 | 0 | 0 | 0 |
| Rubber | BR2b | 2016 | 0 | 0 | 0 | 0 | 0 | 0 | 40 | 32 | 0 | 0 | 0 | 0 | 0 | 4 | 0 | 0 | 0 | 0 | 0 | 0 | 0 |
| Rubber | BR3b | 2016 | 0 | 0 | 0 | 0 | 8 | 0 | 32 | 8 | 4 | 0 | 0 | 0 | 0 | 0 | 0 | 0 | 0 | 0 | 0 | 0 | 12 |
| Rubber | BR4b | 2016 | 4 | 0 | 0 | 0 | 0 | 0 | 0 | 0 | 8 | 0 | 0 | 0 | 0 | 0 | 4 | 4 | 0 | 0 | 0 | 0 | 0 |
| Rubber | HR2b | 2016 | 0 | 0 | 0 | 0 | 0 | 0 | 0 | 0 | 8 | 0 | 0 | 0 | 0 | 0 | 0 | 0 | 0 | 0 | 0 | 0 | 0 |
| Rubber | HR3b | 2016 | 0 | 0 | 4 | 0 | 8 | 0 | 0 | 0 | 0 | 8 | 0 | 0 | 0 | 0 | 0 | 0 | 0 | 0 | 0 | 0 | 0 |
| Rubber | HR4b | 2016 | 0 | 0 | 0 | 0 | 4 | 0 | 4 | 0 | 4 | 4 | 0 | 0 | 0 | 0 | 0 | 0 | 0 | 0 | 0 | 0 | 0 |
| Systems | Plot | Year | *Acr1* | *Acr2* | *Allo* | *Call1* | *Asco* | *Dicr1* | *Folce* | *Folspa* | *Homci* | *Isomi* | *Lep1* | *Mega* | *Ony* | *Par1* | *Pseu1* | *Ram* | *Spha1* | *Spha2* | *Sphy1* | *Sup1* | *Xen1* |
| Rubber | HRr2b | 2016 | 0 | 0 | 0 | 0 | 12 | 0 | 12 | 0 | 0 | 8 | 0 | 0 | 0 | 0 | 0 | 0 | 0 | 0 | 0 | 0 | 8 |
| Rubber | HRr3b | 2016 | 0 | 0 | 0 | 0 | 12 | 0 | 0 | 0 | 8 | 0 | 0 | 0 | 0 | 0 | 0 | 0 | 0 | 0 | 0 | 0 | 0 |
| Rubber | HRr4b | 2016 | 0 | 0 | 0 | 0 | 16 | 0 | 8 | 12 | 0 | 0 | 0 | 0 | 0 | 0 | 0 | 0 | 0 | 0 | 0 | 0 | 0 |

Species matrix of Collembola in the litter layer (species present in at least two plots in every land-use system). *Acr1=Acrocyrtus sp.1, Acr2=Acrocyrtus sp.2, Allo=Alloscopus tetracanthus, Call1=Callyntrura sp.1, Asco=Ascocyrtus cinctus, Dicr1=Dicranocentrus sp.1, Folce=Folsomides centralis, Folspa=Folsomides parvulus, Homci=Homidia cingula, Isomi=Isotomiella spp., Lep1=Lepidocyrtus sp.1, Mega=Megalothorax cf.minimus, Ony=Onychiuridae spp., Par1=Pararrhopalites sp.1, Pseu1=Pseudosinella sp.1, Ram=Rambutsinella sp.1, Spha1= Sphaeridia sp.1, Spha2=Sphaeridia sp.2, Sphy1= Sphyroteca sp.1, Sup1=Superodontella sp.1, Xen1= Xenylla sp.1*

**Supplementary Table S3. Species matrix of Collembola (individuals per plot) in the soil layer**

| Systems | Plot | Year | *Acr1* | *Acr3* | *Allo* | *Asco* | *Cyps1* | *Cyph1* | *Dicr1* | *Folsce* | *Folspa* | *Folny* | *Homci* | *Isomi* | *Isode1* | *Isotus* | *Lep1* | *Mega* | *Ony* | *Pseu1* | *Ram* | *Sphy1* | *Xen1* |
| --- | --- | --- | --- | --- | --- | --- | --- | --- | --- | --- | --- | --- | --- | --- | --- | --- | --- | --- | --- | --- | --- | --- | --- |
| Rainforest | BF1b | 2013 | 2 | 0 | 0 | 0 | 10 | 0 | 0 | 0 | 0 | 0 | 0 | 5 | 0 | 0 | 2 | 0 | 0 | 12 | 0 | 0 | 0 |
| Rainforest | BF2b | 2013 | 0 | 3 | 1 | 1 | 0 | 0 | 0 | 8 | 10 | 0 | 0 | 17 | 0 | 0 | 1 | 0 | 15 | 6 | 0 | 1 | 0 |
| Rainforest | BF3b | 2013 | 0 | 0 | 0 | 0 | 2 | 0 | 0 | 0 | 0 | 1 | 0 | 0 | 0 | 0 | 0 | 0 | 0 | 0 | 0 | 0 | 0 |
| Rainforest | BF4b | 2013 | 0 | 2 | 0 | 0 | 7 | 0 | 0 | 1 | 0 | 0 | 0 | 385 | 0 | 0 | 0 | 3 | 93 | 54 | 2 | 0 | 1 |
| Rainforest | HF1b | 2013 | 0 | 0 | 0 | 0 | 0 | 0 | 0 | 1 | 0 | 0 | 1 | 34 | 0 | 0 | 4 | 0 | 0 | 39 | 0 | 0 | 1 |
| Rainforest | HF2b | 2013 | 0 | 0 | 0 | 0 | 0 | 0 | 1 | 0 | 0 | 0 | 0 | 41 | 0 | 0 | 6 | 0 | 0 | 39 | 0 | 0 | 2 |
| Rainforest | HF3b | 2013 | 0 | 0 | 0 | 0 | 0 | 0 | 0 | 0 | 0 | 0 | 0 | 0 | 0 | 0 | 11 | 0 | 0 | 28 | 1 | 0 | 0 |
| Rainforest | HF4b | 2013 | 0 | 0 | 0 | 0 | 0 | 0 | 0 | 0 | 0 | 0 | 0 | 5 | 0 | 0 | 7 | 0 | 0 | 56 | 16 | 0 | 8 |
| Rainforest | BF1b | 2016 | 0 | 0 | 0 | 0 | 0 | 0 | 0 | 0 | 0 | 0 | 0 | 4 | 0 | 0 | 0 | 0 | 0 | 4 | 0 | 0 | 0 |
| Rainforest | BF2b | 2016 | 0 | 0 | 16 | 0 | 0 | 0 | 0 | 4 | 0 | 0 | 0 | 12 | 0 | 0 | 0 | 0 | 0 | 0 | 8 | 0 | 12 |
| Rainforest | BF3b | 2016 | 0 | 0 | 4 | 4 | 0 | 0 | 0 | 0 | 0 | 0 | 0 | 4 | 0 | 0 | 0 | 0 | 0 | 8 | 0 | 0 | 0 |
| Rainforest | BF4b | 2016 | 0 | 0 | 0 | 0 | 0 | 0 | 0 | 0 | 0 | 0 | 0 | 4 | 0 | 0 | 0 | 0 | 12 | 8 | 0 | 0 | 0 |
| Rainforest | HF1b | 2016 | 0 | 0 | 12 | 0 | 0 | 0 | 0 | 0 | 0 | 0 | 0 | 4 | 0 | 4 | 0 | 0 | 0 | 24 | 0 | 0 | 0 |
| Rainforest | HF2b | 2016 | 0 | 0 | 12 | 4 | 0 | 0 | 0 | 0 | 0 | 0 | 0 | 0 | 0 | 0 | 0 | 0 | 0 | 0 | 0 | 0 | 0 |
| Rainforest | HF3b | 2016 | 0 | 0 | 44 | 0 | 0 | 8 | 0 | 16 | 4 | 0 | 0 | 120 | 0 | 0 | 0 | 0 | 0 | 8 | 4 | 0 | 0 |
| Rainforest | HF4b | 2016 | 0 | 0 | 8 | 32 | 4 | 4 | 0 | 0 | 0 | 0 | 0 | 88 | 0 | 0 | 0 | 0 | 16 | 8 | 16 | 12 | 12 |
| Rainforest | HFr1b | 2016 | 0 | 0 | 0 | 0 | 0 | 0 | 0 | 0 | 0 | 0 | 4 | 0 | 0 | 0 | 0 | 0 | 0 | 0 | 0 | 0 | 0 |
| Rainforest | HFr2b | 2016 | 0 | 0 | 4 | 8 | 16 | 0 | 0 | 0 | 0 | 0 | 0 | 4 | 0 | 0 | 0 | 0 | 0 | 4 | 0 | 0 | 0 |
| Rainforest | HFr3b | 2016 | 0 | 0 | 0 | 8 | 0 | 0 | 0 | 4 | 0 | 0 | 0 | 0 | 0 | 0 | 0 | 0 | 0 | 0 | 0 | 0 | 0 |
| Rainforest | HFr4b | 2016 | 0 | 0 | 0 | 4 | 0 | 0 | 0 | 0 | 0 | 0 | 0 | 8 | 0 | 0 | 0 | 0 | 0 | 0 | 0 | 0 | 0 |
| Jungle rubber | BJ2b | 2013 | 0 | 0 | 2 | 0 | 0 | 2 | 0 | 11 | 1 | 0 | 1 | 2 | 0 | 0 | 0 | 0 | 0 | 20 | 3 | 0 | 0 |
| Jungle rubber | BJ3b | 2013 | 0 | 0 | 0 | 8 | 0 | 1 | 0 | 12 | 1 | 0 | 3 | 12 | 0 | 0 | 0 | 0 | 0 | 16 | 2 | 0 | 0 |
| Jungle rubber | BJ4b | 2013 | 0 | 0 | 0 | 0 | 0 | 0 | 0 | 32 | 4 | 2 | 0 | 0 | 0 | 0 | 0 | 0 | 0 | 0 | 0 | 0 | 0 |
| Jungle rubber | BJ5b | 2013 | 0 | 0 | 1 | 3 | 0 | 0 | 0 | 3 | 0 | 0 | 1 | 1 | 0 | 0 | 0 | 1 | 0 | 26 | 25 | 1 | 1 |
| Jungle rubber | HJ1b | 2013 | 0 | 0 | 0 | 3 | 0 | 0 | 0 | 12 | 5 | 0 | 0 | 6 | 0 | 0 | 0 | 0 | 0 | 0 | 2 | 0 | 0 |
| Jungle rubber | HJ3b | 2013 | 0 | 0 | 0 | 1 | 0 | 0 | 0 | 0 | 0 | 0 | 0 | 2 | 6 | 0 | 0 | 0 | 0 | 3 | 0 | 0 | 0 |
| Jungle rubber | HJ4b | 2013 | 0 | 0 | 0 | 1 | 0 | 0 | 0 | 0 | 2 | 1 | 0 | 0 | 0 | 0 | 0 | 0 | 6 | 7 | 0 | 0 | 0 |
| Oil palm | BO2b | 2013 | 0 | 0 | 0 | 0 | 0 | 1 | 0 | 13 | 0 | 0 | 0 | 7 | 0 | 0 | 2 | 0 | 0 | 47 | 0 | 0 | 0 |
| Systems | Plot | Year | *Acr1* | *Acr3* | *Allo* | *Asco* | *Cyps1* | *Cyph1* | *Dicr1* | *Folsce* | *Folspa* | *Folny* | *Homci* | *Isomi* | *Isode1* | *Isotus* | *Lep1* | *Mega* | *Ony* | *Pseu1* | *Ram* | *Sphy1* | *Xen1* |
| Oil palm | BO3b | 2013 | 0 | 0 | 0 | 2 | 0 | 0 | 0 | 5 | 0 | 0 | 7 | 14 | 0 | 0 | 0 | 1 | 16 | 14 | 4 | 0 | 0 |
| Oil palm | BO4b | 2013 | 0 | 0 | 9 | 0 | 0 | 0 | 0 | 57 | 0 | 0 | 0 | 91 | 0 | 2 | 2 | 1 | 5 | 38 | 1 | 0 | 0 |
| Oil palm | HO1b | 2013 | 0 | 5 | 20 | 0 | 0 | 0 | 0 | 21 | 0 | 0 | 0 | 37 | 0 | 0 | 7 | 0 | 0 | 41 | 0 | 0 | 0 |
| Oil palm | HO2b | 2013 | 0 | 2 | 0 | 0 | 0 | 0 | 13 | 23 | 0 | 0 | 0 | 21 | 0 | 0 | 1 | 0 | 1 | 11 | 0 | 0 | 0 |
| Oil palm | HO3b | 2013 | 0 | 0 | 0 | 0 | 0 | 0 | 0 | 0 | 0 | 0 | 0 | 11 | 0 | 0 | 1 | 0 | 0 | 10 | 0 | 0 | 0 |
| Oil palm | BO1b | 2016 | 4 | 0 | 16 | 0 | 0 | 0 | 0 | 8 | 0 | 0 | 0 | 0 | 0 | 0 | 0 | 0 | 0 | 4 | 36 | 0 | 0 |
| Oil palm | BO2b | 2016 | 16 | 0 | 12 | 0 | 0 | 0 | 0 | 16 | 0 | 0 | 0 | 8 | 8 | 0 | 0 | 0 | 0 | 4 | 12 | 0 | 0 |
| Oil palm | BO3b | 2016 | 0 | 0 | 4 | 12 | 0 | 0 | 0 | 8 | 0 | 0 | 0 | 0 | 0 | 0 | 0 | 0 | 0 | 8 | 0 | 0 | 0 |
| Oil palm | BO4b | 2016 | 0 | 0 | 16 | 0 | 0 | 0 | 12 | 12 | 0 | 0 | 0 | 0 | 12 | 0 | 0 | 0 | 0 | 28 | 0 | 0 | 0 |
| Oil palm | HO2b | 2016 | 0 | 0 | 0 | 4 | 0 | 0 | 0 | 8 | 0 | 0 | 0 | 0 | 0 | 8 | 0 | 0 | 0 | 0 | 4 | 0 | 0 |
| Oil palm | HO3b | 2016 | 4 | 0 | 0 | 0 | 0 | 0 | 0 | 8 | 0 | 20 | 4 | 4 | 4 | 0 | 0 | 0 | 8 | 0 | 0 | 0 | 0 |
| Oil palm | HO4b | 2016 | 0 | 0 | 16 | 0 | 4 | 0 | 0 | 4 | 0 | 16 | 0 | 0 | 0 | 0 | 0 | 0 | 4 | 0 | 4 | 0 | 0 |
| Oil palm | HO4rb | 2016 | 0 | 0 | 0 | 4 | 0 | 0 | 0 | 8 | 4 | 0 | 0 | 60 | 0 | 0 | 0 | 0 | 12 | 32 | 64 | 0 | 0 |
| Oil palm | HOr1b | 2016 | 0 | 0 | 24 | 0 | 0 | 0 | 0 | 16 | 0 | 0 | 0 | 4 | 0 | 0 | 0 | 0 | 0 | 0 | 0 | 0 | 0 |
| Oil palm | HOr3b | 2016 | 0 | 0 | 24 | 0 | 0 | 0 | 0 | 20 | 0 | 0 | 0 | 0 | 0 | 4 | 0 | 0 | 0 | 16 | 0 | 0 | 0 |
| Rubber | BR1b | 2013 | 0 | 0 | 6 | 22 | 1 | 0 | 0 | 5 | 1 | 0 | 0 | 22 | 0 | 0 | 2 | 2 | 0 | 2 | 4 | 0 | 0 |
| Rubber | BR2b | 2013 | 3 | 1 | 0 | 0 | 0 | 0 | 0 | 16 | 5 | 0 | 0 | 3 | 0 | 0 | 0 | 2 | 0 | 14 | 0 | 0 | 0 |
| Rubber | BR3b | 2013 | 2 | 0 | 0 | 0 | 0 | 0 | 0 | 7 | 0 | 0 | 1 | 0 | 0 | 0 | 0 | 0 | 0 | 15 | 25 | 0 | 0 |
| Rubber | BR4b | 2013 | 0 | 0 | 0 | 0 | 0 | 0 | 0 | 4 | 0 | 0 | 0 | 0 | 1 | 0 | 0 | 0 | 0 | 8 | 0 | 0 | 0 |
| Rubber | HR1b | 2013 | 0 | 4 | 17 | 6 | 0 | 1 | 0 | 7 | 0 | 0 | 0 | 20 | 0 | 0 | 0 | 0 | 0 | 35 | 0 | 2 | 0 |
| Rubber | HR2b | 2013 | 0 | 0 | 5 | 18 | 5 | 0 | 0 | 2 | 0 | 0 | 0 | 0 | 0 | 0 | 0 | 6 | 0 | 0 | 0 | 0 | 0 |
| Rubber | HR3b | 2013 | 2 | 0 | 6 | 0 | 0 | 0 | 0 | 6 | 4 | 0 | 0 | 0 | 0 | 0 | 0 | 0 | 0 | 15 | 0 | 0 | 0 |
| Rubber | HR4b | 2013 | 0 | 0 | 2 | 0 | 0 | 0 | 0 | 0 | 0 | 0 | 0 | 0 | 1 | 0 | 0 | 0 | 0 | 20 | 0 | 0 | 0 |
| Rubber | BR1b | 2016 | 0 | 0 | 0 | 8 | 0 | 0 | 0 | 0 | 0 | 0 | 0 | 8 | 0 | 0 | 0 | 0 | 0 | 0 | 4 | 0 | 0 |
| Rubber | BR2b | 2016 | 0 | 0 | 28 | 0 | 0 | 0 | 0 | 16 | 4 | 0 | 0 | 0 | 0 | 0 | 0 | 0 | 0 | 24 | 0 | 0 | 0 |
| Rubber | BR3b | 2016 | 0 | 0 | 0 | 0 | 0 | 0 | 0 | 16 | 0 | 0 | 0 | 0 | 0 | 0 | 0 | 0 | 0 | 4 | 0 | 0 | 0 |
| Rubber | BR4b | 2016 | 0 | 0 | 20 | 0 | 0 | 0 | 0 | 0 | 0 | 0 | 0 | 0 | 0 | 0 | 0 | 0 | 0 | 4 | 32 | 0 | 0 |
| Rubber | HR1b | 2016 | 0 | 0 | 0 | 12 | 0 | 0 | 0 | 4 | 0 | 0 | 0 | 0 | 0 | 0 | 0 | 0 | 0 | 40 | 0 | 0 | 0 |
| Rubber | HR2b | 2016 | 0 | 0 | 36 | 44 | 0 | 0 | 0 | 12 | 0 | 0 | 0 | 0 | 0 | 0 | 0 | 0 | 0 | 0 | 4 | 0 | 0 |
| Rubber | HR3b | 2016 | 0 | 0 | 4 | 8 | 0 | 8 | 0 | 12 | 0 | 0 | 4 | 0 | 0 | 0 | 0 | 0 | 0 | 16 | 0 | 0 | 0 |
| Systems | Plot | Year | *Acr1* | *Acr3* | *Allo* | *Asco* | *Cyps1* | *Cyph1* | *Dicr1* | *Folsce* | *Folspa* | *Folny* | *Homci* | *Isomi* | *Isode1* | *Isotus* | *Lep1* | *Mega* | *Ony* | *Pseu1* | *Ram* | *Sphy1* | *Xen1* |
| Rubber | HR4b | 2016 | 0 | 0 | 0 | 12 | 0 | 8 | 0 | 0 | 0 | 0 | 0 | 0 | 0 | 0 | 0 | 0 | 0 | 0 | 4 | 0 | 0 |
| Rubber | HRr1b | 2016 | 0 | 0 | 12 | 8 | 0 | 0 | 0 | 4 | 0 | 0 | 8 | 0 | 0 | 8 | 0 | 0 | 0 | 0 | 0 | 0 | 0 |
| Rubber | HRr2b | 2016 | 0 | 0 | 4 | 8 | 0 | 0 | 0 | 0 | 0 | 0 | 0 | 0 | 0 | 0 | 0 | 0 | 0 | 0 | 0 | 0 | 0 |
| Rubber | HRr3b | 2016 | 0 | 0 | 4 | 12 | 0 | 4 | 0 | 12 | 0 | 0 | 12 | 0 | 0 | 0 | 0 | 0 | 0 | 20 | 0 | 0 | 0 |
| Rubber | HRr4b | 2016 | 0 | 0 | 0 | 16 | 0 | 0 | 0 | 4 | 8 | 4 | 12 | 8 | 0 | 0 | 0 | 0 | 0 | 0 | 0 | 0 | 0 |

Species matrix of Collembola in the soil layer (species present in at least two plots in every land-use system). *Acr1=Acrocyrtus sp.1, Acr3=Acrocyrtus sp.3, Allo=Alloscopus tetracanthus, Asco=Ascocyrtus cinctus, Cyps1= Cyphoderopsis sp.1, Cyph=Cyphoderus sp.1, Dicr1=Dicranocentrus sp.1, Folce=Folsomides centralis, Folspa=Folsomides parvulus, Homci=Homidia cingula, Isomi=Isotomiella spp., Isode1=Isotomodes sp.1, Isotus=Isotomurus cf. Parbalteatus, Lep1=Lepidocyrtus sp.1, Mega=Megalothorax cf.minimus, Ony=Onychiuridae spp., Pseu1=Pseudosinella sp.1, Ram=Rambutsinella sp.1, Sphy1= Sphyroteca sp.1, Xen1= Xenylla sp.1*

**Supplementary Table S4. Trait matrix of Collembola in litter layer based on density**

| System | Plot | Year | Ea | Ms | PAOp | Sp | Ae | As | Af | Fs | Fa | Fl | Pd | Pp | Pa | Pi | Am | Ss | Ms | Ls |
| --- | --- | --- | --- | --- | --- | --- | --- | --- | --- | --- | --- | --- | --- | --- | --- | --- | --- | --- | --- | --- |
| Rainforest | BF1b | 2013 | 1 | 1 | 22 | 37 | 28 | 6 | 21 | 26 | 2 | 58 | 4 | 37 | 44 | 1 | 18 | 50 | 22 | 14 |
| Rainforest | BF2b | 2013 | 28 | 0 | 51 | 0 | 0 | 1 | 2 | 22 | 30 | 2 | 21 | 0 | 32 | 1 | 0 | 54 | 0 | 0 |
| Rainforest | BF3b | 2013 | 5 | 2 | 18 | 43 | 50 | 10 | 52 | 70 | 13 | 60 | 21 | 36 | 81 | 5 | 17 | 39 | 32 | 72 |
| Rainforest | BF4b | 2013 | 1 | 0 | 18 | 15 | 11 | 14 | 28 | 15 | 17 | 43 | 6 | 22 | 46 | 1 | 17 | 59 | 16 | 0 |
| Rainforest | HF1b | 2013 | 5 | 0 | 15 | 44 | 32 | 37 | 15 | 51 | 0 | 59 | 32 | 32 | 35 | 11 | 38 | 66 | 38 | 6 |
| Rainforest | HF2b | 2013 | 0 | 0 | 1 | 0 | 0 | 0 | 0 | 0 | 1 | 0 | 0 | 0 | 1 | 0 | 0 | 1 | 0 | 0 |
| Rainforest | HF3b | 2013 | 0 | 0 | 2 | 35 | 35 | 25 | 3 | 25 | 2 | 38 | 17 | 21 | 24 | 3 | 21 | 30 | 35 | 0 |
| Rainforest | HF4b | 2013 | 2 | 2 | 2 | 57 | 53 | 28 | 6 | 22 | 0 | 71 | 57 | 25 | 7 | 4 | 24 | 34 | 56 | 3 |
| Rainforest | BF1b | 2016 | 4 | 4 | 16 | 104 | 96 | 0 | 16 | 8 | 0 | 120 | 8 | 24 | 92 | 4 | 20 | 24 | 96 | 8 |
| Rainforest | BF2b | 2016 | 0 | 0 | 44 | 20 | 20 | 12 | 36 | 56 | 0 | 56 | 16 | 8 | 76 | 12 | 8 | 92 | 12 | 8 |
| Rainforest | BF3b | 2016 | 4 | 0 | 20 | 0 | 0 | 0 | 0 | 16 | 4 | 0 | 16 | 0 | 4 | 0 | 0 | 20 | 0 | 0 |
| Rainforest | HF1b | 2016 | 0 | 0 | 0 | 0 | 0 | 0 | 12 | 0 | 0 | 12 | 0 | 0 | 12 | 0 | 0 | 12 | 0 | 0 |
| Rainforest | HF2b | 2016 | 28 | 12 | 12 | 36 | 36 | 4 | 8 | 40 | 0 | 36 | 16 | 16 | 32 | 12 | 4 | 40 | 36 | 0 |
| Rainforest | HF3b | 2016 | 4 | 0 | 40 | 96 | 92 | 0 | 68 | 40 | 0 | 164 | 72 | 36 | 96 | 0 | 4 | 108 | 92 | 4 |
| Rainforest | HFr1b | 2016 | 0 | 0 | 0 | 8 | 8 | 4 | 4 | 4 | 0 | 12 | 0 | 12 | 4 | 0 | 4 | 8 | 8 | 0 |
| Rainforest | HFr2b | 2016 | 16 | 0 | 24 | 32 | 24 | 20 | 84 | 52 | 0 | 116 | 32 | 36 | 100 | 0 | 28 | 132 | 28 | 8 |
| Rainforest | HFr3b | 2016 | 8 | 0 | 4 | 4 | 4 | 0 | 4 | 12 | 0 | 8 | 12 | 4 | 4 | 0 | 0 | 16 | 4 | 0 |
| Rainforest | HFr4b | 2016 | 4 | 4 | 4 | 8 | 8 | 4 | 0 | 8 | 0 | 8 | 0 | 8 | 4 | 4 | 4 | 8 | 8 | 0 |
| Jungle rubber | BJ2b | 2013 | 0 | 0 | 23 | 18 | 18 | 27 | 2 | 50 | 0 | 20 | 23 | 18 | 5 | 24 | 2 | 50 | 18 | 2 |
| Jungle rubber | BJ3b | 2013 | 0 | 0 | 106 | 1 | 10 | 13 | 7 | 118 | 0 | 18 | 98 | 10 | 15 | 13 | 1 | 125 | 0 | 11 |
| Jungle rubber | BJ4b | 2013 | 0 | 0 | 37 | 0 | 0 | 0 | 4 | 37 | 0 | 4 | 21 | 0 | 20 | 0 | 0 | 41 | 0 | 0 |
| Jungle rubber | BJ5b | 2013 | 1 | 0 | 23 | 0 | 0 | 0 | 1 | 25 | 0 | 0 | 13 | 0 | 12 | 0 | 0 | 24 | 0 | 1 |
| Jungle rubber | HJ1b | 2013 | 0 | 0 | 149 | 15 | 15 | 5 | 3 | 151 | 0 | 21 | 71 | 17 | 84 | 0 | 3 | 158 | 9 | 5 |
| Jungle rubber | HJ2b | 2013 | 0 | 0 | 19 | 21 | 39 | 0 | 32 | 20 | 0 | 70 | 15 | 24 | 51 | 0 | 0 | 51 | 20 | 19 |
| Jungle rubber | HJ3b | 2013 | 0 | 0 | 1 | 2 | 2 | 2 | 0 | 3 | 0 | 2 | 1 | 3 | 1 | 0 | 2 | 3 | 2 | 0 |
| Jungle rubber | HJ4b | 2013 | 0 | 0 | 31 | 1 | 1 | 7 | 10 | 38 | 0 | 11 | 13 | 5 | 28 | 3 | 4 | 48 | 1 | 0 |
| Oil palm | BO2b | 2013 | 3 | 0 | 4 | 2 | 2 | 1 | 0 | 1 | 4 | 2 | 2 | 0 | 4 | 1 | 0 | 5 | 2 | 0 |
| Oil palm | BO3b | 2013 | 1 | 0 | 41 | 17 | 11 | 8 | 0 | 41 | 5 | 17 | 36 | 8 | 19 | 0 | 12 | 47 | 12 | 4 |
| Oil palm | BO4b | 2013 | 0 | 0 | 0 | 6 | 6 | 2 | 6 | 2 | 0 | 12 | 3 | 1 | 9 | 1 | 1 | 7 | 7 | 0 |
| Oil palm | BO5b | 2013 | 0 | 0 | 21 | 5 | 5 | 0 | 3 | 18 | 3 | 8 | 12 | 0 | 17 | 0 | 0 | 24 | 5 | 0 |
| Oil palm | HO1b | 2013 | 0 | 0 | 25 | 7 | 7 | 1 | 0 | 28 | 0 | 5 | 25 | 3 | 5 | 0 | 1 | 25 | 6 | 2 |
| Oil palm | HO2b | 2013 | 1 | 1 | 1 | 1 | 1 | 0 | 10 | 1 | 0 | 11 | 1 | 0 | 10 | 1 | 0 | 10 | 2 | 0 |
| Oil palm | HO3b | 2013 | 1 | 0 | 1 | 1 | 2 | 0 | 13 | 1 | 1 | 15 | 2 | 0 | 15 | 0 | 0 | 15 | 2 | 0 |
| Oil palm | BO1b | 2016 | 0 | 0 | 28 | 208 | 200 | 8 | 60 | 40 | 0 | 260 | 204 | 4 | 84 | 8 | 8 | 84 | 204 | 12 |
| Oil palm | BO2b | 2016 | 0 | 0 | 4 | 4 | 8 | 0 | 0 | 4 | 0 | 8 | 8 | 4 | 0 | 0 | 0 | 4 | 4 | 4 |
| Oil palm | BO3b | 2016 | 0 | 0 | 24 | 4 | 4 | 0 | 0 | 24 | 0 | 4 | 12 | 0 | 16 | 0 | 0 | 24 | 4 | 0 |
| Oil palm | BO4b | 2016 | 0 | 0 | 48 | 4 | 4 | 0 | 0 | 48 | 0 | 4 | 48 | 0 | 4 | 0 | 0 | 48 | 4 | 0 |
| Oil palm | HO1b | 2016 | 0 | 0 | 0 | 0 | 4 | 0 | 0 | 0 | 0 | 4 | 4 | 0 | 0 | 0 | 0 | 4 | 0 | 0 |
| Oil palm | HO2b | 2016 | 8 | 0 | 24 | 0 | 0 | 0 | 0 | 16 | 8 | 0 | 16 | 0 | 8 | 0 | 0 | 24 | 0 | 0 |
| Oil palm | HO3b | 2016 | 0 | 0 | 4 | 8 | 8 | 0 | 4 | 8 | 0 | 8 | 12 | 0 | 4 | 0 | 0 | 4 | 0 | 12 |
| Oil palm | HO4b | 2016 | 0 | 0 | 4 | 8 | 8 | 0 | 0 | 4 | 0 | 8 | 12 | 0 | 0 | 0 | 0 | 4 | 8 | 0 |
| Oil palm | HO4rb | 2016 | 0 | 0 | 0 | 0 | 0 | 0 | 20 | 0 | 0 | 20 | 0 | 0 | 20 | 0 | 0 | 20 | 0 | 0 |
| Oil palm | HOr2b | 2016 | 0 | 0 | 20 | 0 | 0 | 0 | 0 | 12 | 0 | 8 | 12 | 8 | 0 | 0 | 0 | 12 | 8 | 0 |
| Oil palm | HOr3b | 2016 | 0 | 0 | 24 | 0 | 0 | 0 | 0 | 24 | 0 | 0 | 20 | 0 | 4 | 0 | 0 | 24 | 0 | 0 |
| Rubber | BR1b | 2013 | 0 | 0 | 14 | 27 | 28 | 6 | 8 | 20 | 0 | 36 | 17 | 18 | 21 | 0 | 1 | 27 | 28 | 1 |
| Rubber | BR2b | 2013 | 0 | 0 | 7 | 9 | 9 | 3 | 0 | 10 | 0 | 9 | 7 | 9 | 3 | 0 | 3 | 10 | 9 | 0 |
| Rubber | BR3b | 2013 | 0 | 0 | 17 | 41 | 56 | 2 | 5 | 19 | 0 | 61 | 23 | 39 | 18 | 0 | 2 | 24 | 41 | 15 |
| Rubber | HR1b | 2013 | 0 | 0 | 46 | 33 | 34 | 4 | 18 | 50 | 0 | 52 | 50 | 26 | 26 | 0 | 0 | 68 | 33 | 1 |
| Rubber | HR2b | 2013 | 0 | 0 | 20 | 20 | 33 | 12 | 0 | 36 | 0 | 29 | 20 | 28 | 13 | 4 | 1 | 32 | 20 | 13 |
| Rubber | HR3b | 2013 | 0 | 0 | 10 | 4 | 4 | 0 | 0 | 9 | 0 | 5 | 8 | 1 | 5 | 0 | 1 | 9 | 3 | 2 |
| Rubber | HR4b | 2013 | 0 | 0 | 6 | 3 | 3 | 0 | 0 | 6 | 0 | 3 | 2 | 2 | 5 | 0 | 0 | 6 | 3 | 0 |
| Rubber | BR1b | 2016 | 0 | 0 | 4 | 8 | 8 | 0 | 0 | 4 | 0 | 8 | 4 | 8 | 0 | 0 | 0 | 4 | 8 | 0 |
| Rubber | BR2b | 2016 | 0 | 0 | 72 | 0 | 0 | 4 | 0 | 76 | 0 | 0 | 40 | 4 | 32 | 0 | 4 | 76 | 0 | 0 |
| Rubber | BR3b | 2016 | 12 | 0 | 40 | 8 | 12 | 0 | 0 | 52 | 0 | 12 | 44 | 12 | 8 | 0 | 0 | 52 | 8 | 4 |
| Rubber | BR4b | 2016 | 0 | 0 | 0 | 12 | 20 | 0 | 0 | 0 | 0 | 20 | 4 | 8 | 8 | 0 | 0 | 0 | 12 | 8 |
| Rubber | HR2b | 2016 | 0 | 0 | 0 | 0 | 8 | 0 | 0 | 0 | 0 | 8 | 0 | 8 | 0 | 0 | 0 | 0 | 0 | 8 |
| Rubber | HR3b | 2016 | 0 | 0 | 4 | 12 | 8 | 0 | 8 | 0 | 0 | 20 | 0 | 8 | 12 | 0 | 4 | 8 | 8 | 4 |
| Rubber | HR4b | 2016 | 0 | 0 | 4 | 4 | 8 | 0 | 4 | 4 | 0 | 12 | 4 | 8 | 4 | 0 | 0 | 8 | 4 | 4 |
| Rubber | HRr1b | 2016 | 0 | 0 | 48 | 84 | 48 | 0 | 0 | 12 | 0 | 84 | 16 | 44 | 36 | 0 | 36 | 12 | 48 | 36 |
| Rubber | HRr2b | 2016 | 8 | 0 | 12 | 12 | 12 | 0 | 8 | 20 | 0 | 20 | 20 | 12 | 8 | 0 | 0 | 28 | 12 | 0 |
| Rubber | HRr3b | 2016 | 0 | 0 | 0 | 12 | 20 | 0 | 0 | 0 | 0 | 20 | 0 | 20 | 0 | 0 | 0 | 0 | 12 | 8 |
| Rubber | HRr4b | 2016 | 0 | 0 | 20 | 16 | 16 | 0 | 0 | 20 | 0 | 16 | 8 | 16 | 12 | 0 | 0 | 20 | 16 | 0 |

Abbreviation of traits: Ea=Empodial appendage absent, Ms=Mouthpart sucking, PAOp= PAO present, Sp=Scales present, Ae=Abdoment IV elongated,

As=Abdoment spherical, Af=Abdoment fused, Fs=Furca straight, Fa=Furca absent, Fl=Furca long, Pd=Pigmenttaion diffuse, Pp=Pigmentation pattern,

Pa=Pigmentation absent, Pi=Pigmenttaion intensive, Am=Antennae modified, Ss=Small size, Ms=Medium size, Ls=Large size

**Supplementary Table S5. Trait matrix of Collembola in soil layer based on density**

| System | Plot | Year | Ea | Ms | PAOp | Sp | Ae | As | Af | Fs | Fa | Fl | Pd | Pp | Pa | Pi | Am | Ss | Ms | Ls |
| --- | --- | --- | --- | --- | --- | --- | --- | --- | --- | --- | --- | --- | --- | --- | --- | --- | --- | --- | --- | --- |
| Rainforest | BF1b | 2013 | 4 | 4 | 2 | 26 | 26 | 0 | 5 | 3 | 1 | 31 | 2 | 0 | 29 | 4 | 0 | 5 | 29 | 1 |
| Rainforest | BF2b | 2013 | 0 | 0 | 34 | 12 | 11 | 2 | 17 | 20 | 15 | 29 | 9 | 5 | 49 | 1 | 2 | 52 | 8 | 4 |
| Rainforest | BF3b | 2013 | 0 | 0 | 0 | 2 | 2 | 0 | 2 | 2 | 0 | 2 | 0 | 0 | 4 | 0 | 0 | 0 | 3 | 1 |
| Rainforest | BF4b | 2013 | 1 | 0 | 94 | 66 | 66 | 3 | 385 | 5 | 93 | 451 | 5 | 2 | 542 | 0 | 0 | 483 | 64 | 2 |
| Rainforest | HF1b | 2013 | 1 | 0 | 1 | 43 | 44 | 1 | 34 | 3 | 0 | 78 | 6 | 2 | 73 | 0 | 1 | 37 | 43 | 1 |
| Rainforest | HF2b | 2013 | 3 | 1 | 1 | 52 | 51 | 2 | 41 | 5 | 0 | 93 | 14 | 3 | 80 | 1 | 3 | 45 | 53 | 0 |
| Rainforest | HF3b | 2013 | 0 | 0 | 0 | 40 | 40 | 1 | 0 | 1 | 0 | 40 | 12 | 1 | 28 | 0 | 1 | 1 | 40 | 0 |
| Rainforest | HF4b | 2013 | 8 | 0 | 0 | 85 | 85 | 1 | 5 | 9 | 0 | 90 | 37 | 1 | 61 | 0 | 1 | 14 | 85 | 0 |
| Rainforest | BF1b | 2016 | 0 | 0 | 0 | 4 | 4 | 0 | 4 | 0 | 0 | 8 | 0 | 0 | 8 | 0 | 0 | 4 | 4 | 0 |
| Rainforest | BF2b | 2016 | 12 | 0 | 20 | 24 | 8 | 0 | 12 | 16 | 0 | 36 | 24 | 0 | 28 | 0 | 16 | 28 | 8 | 16 |
| Rainforest | BF3b | 2016 | 0 | 0 | 4 | 16 | 12 | 0 | 4 | 0 | 0 | 20 | 0 | 4 | 16 | 0 | 4 | 4 | 12 | 4 |
| Rainforest | BF4b | 2016 | 12 | 0 | 12 | 8 | 8 | 0 | 4 | 0 | 12 | 12 | 0 | 0 | 24 | 0 | 0 | 16 | 8 | 0 |
| Rainforest | HF1b | 2016 | 0 | 0 | 16 | 36 | 24 | 0 | 4 | 0 | 0 | 44 | 0 | 4 | 40 | 0 | 12 | 4 | 28 | 12 |
| Rainforest | HF2b | 2016 | 0 | 0 | 12 | 16 | 4 | 0 | 0 | 0 | 0 | 16 | 0 | 4 | 12 | 0 | 12 | 0 | 4 | 12 |
| Rainforest | HF3b | 2016 | 0 | 0 | 64 | 64 | 20 | 0 | 120 | 28 | 0 | 176 | 20 | 0 | 184 | 0 | 44 | 140 | 20 | 44 |
| Rainforest | HF4b | 2016 | 44 | 16 | 40 | 72 | 64 | 12 | 88 | 44 | 16 | 156 | 28 | 44 | 128 | 16 | 20 | 144 | 64 | 8 |
| Rainforest | HFr1b | 2016 | 0 | 0 | 0 | 0 | 4 | 0 | 0 | 0 | 0 | 4 | 0 | 4 | 0 | 0 | 0 | 0 | 0 | 4 |
| Rainforest | HFr2b | 2016 | 0 | 0 | 4 | 32 | 28 | 0 | 4 | 0 | 0 | 36 | 0 | 8 | 28 | 0 | 4 | 4 | 28 | 4 |
| Rainforest | HFr3b | 2016 | 0 | 0 | 4 | 8 | 8 | 0 | 0 | 4 | 0 | 8 | 4 | 8 | 0 | 0 | 0 | 4 | 8 | 0 |
| Rainforest | HFr4b | 2016 | 0 | 0 | 0 | 4 | 4 | 0 | 8 | 0 | 0 | 12 | 0 | 4 | 8 | 0 | 0 | 8 | 4 | 0 |
| Jungle rubber | BJ2b | 2013 | 0 | 0 | 14 | 27 | 26 | 0 | 2 | 14 | 0 | 28 | 14 | 1 | 27 | 0 | 2 | 14 | 25 | 3 |
| Jungle rubber | BJ3b | 2013 | 2 | 2 | 13 | 27 | 30 | 0 | 12 | 16 | 0 | 41 | 14 | 11 | 30 | 2 | 0 | 25 | 29 | 3 |
| Jungle rubber | BJ4b | 2013 | 0 | 0 | 36 | 0 | 0 | 1 | 2 | 39 | 0 | 0 | 32 | 0 | 6 | 1 | 0 | 37 | 0 | 2 |
| Jungle rubber | BJ5b | 2013 | 2 | 1 | 5 | 55 | 55 | 2 | 1 | 7 | 0 | 57 | 29 | 5 | 29 | 1 | 2 | 8 | 54 | 2 |
| Jungle rubber | HJ1b | 2013 | 0 | 0 | 17 | 5 | 5 | 0 | 6 | 17 | 0 | 11 | 14 | 3 | 11 | 0 | 0 | 23 | 5 | 0 |
| Jungle rubber | HJ3b | 2013 | 0 | 0 | 6 | 4 | 4 | 0 | 2 | 6 | 0 | 6 | 0 | 1 | 11 | 0 | 0 | 8 | 4 | 0 |
| Jungle rubber | HJ4b | 2013 | 6 | 0 | 8 | 9 | 9 | 0 | 1 | 3 | 6 | 9 | 0 | 2 | 16 | 0 | 1 | 8 | 8 | 2 |
| Oil palm | BO2b | 2013 | 4 | 0 | 17 | 52 | 50 | 7 | 7 | 21 | 4 | 58 | 15 | 3 | 59 | 6 | 3 | 26 | 57 | 0 |
| Oil palm | BO3b | 2013 | 0 | 0 | 90 | 71 | 7 | 2 | 7 | 33 | 0 | 80 | 13 | 11 | 89 | 0 | 66 | 47 | 3 | 63 |
| Oil palm | BO4b | 2013 | 4 | 4 | 25 | 25 | 31 | 10 | 14 | 17 | 16 | 48 | 11 | 17 | 45 | 8 | 7 | 47 | 27 | 7 |
| Oil palm | BO5b | 2013 | 0 | 0 | 73 | 50 | 41 | 2 | 91 | 59 | 5 | 143 | 60 | 3 | 144 | 0 | 10 | 155 | 43 | 9 |
| Oil palm | HO1b | 2013 | 0 | 0 | 24 | 30 | 16 | 2 | 21 | 25 | 1 | 53 | 26 | 18 | 33 | 2 | 16 | 49 | 28 | 2 |
| Oil palm | HO2b | 2013 | 0 | 0 | 0 | 11 | 11 | 1 | 11 | 1 | 0 | 22 | 1 | 1 | 21 | 0 | 1 | 11 | 12 | 0 |
| Oil palm | HO3b | 2013 | 0 | 0 | 24 | 60 | 44 | 0 | 0 | 8 | 0 | 60 | 44 | 0 | 24 | 0 | 16 | 8 | 44 | 16 |
| Oil palm | BO2b | 2016 | 0 | 0 | 36 | 44 | 32 | 0 | 8 | 24 | 0 | 52 | 28 | 0 | 48 | 0 | 12 | 32 | 32 | 12 |
| Oil palm | BO3b | 2016 | 0 | 0 | 12 | 24 | 20 | 4 | 0 | 12 | 0 | 24 | 8 | 16 | 12 | 0 | 8 | 8 | 24 | 4 |
| Oil palm | BO4b | 2016 | 0 | 0 | 40 | 56 | 28 | 0 | 0 | 24 | 0 | 56 | 12 | 12 | 56 | 0 | 28 | 24 | 40 | 16 |
| Oil palm | BO1b | 2016 | 0 | 0 | 16 | 0 | 0 | 0 | 0 | 0 | 0 | 16 | 0 | 16 | 0 | 0 | 0 | 0 | 16 | 0 |
| Oil palm | HO2b | 2016 | 0 | 0 | 16 | 8 | 8 | 0 | 0 | 8 | 0 | 16 | 12 | 12 | 0 | 0 | 0 | 8 | 16 | 0 |
| Oil palm | HO3b | 2016 | 8 | 0 | 20 | 4 | 8 | 0 | 24 | 32 | 8 | 12 | 8 | 4 | 40 | 0 | 0 | 24 | 4 | 24 |
| Oil palm | HO4b | 2016 | 4 | 0 | 24 | 24 | 8 | 0 | 16 | 20 | 4 | 24 | 8 | 0 | 40 | 0 | 16 | 8 | 8 | 32 |
| Oil palm | HO4rb | 2016 | 12 | 0 | 24 | 100 | 100 | 0 | 60 | 12 | 12 | 160 | 72 | 4 | 108 | 0 | 0 | 84 | 100 | 0 |
| Oil palm | HOr1b | 2016 | 0 | 0 | 40 | 24 | 0 | 0 | 4 | 16 | 0 | 28 | 16 | 0 | 28 | 0 | 24 | 20 | 0 | 24 |
| Oil palm | HOr3b | 2016 | 0 | 0 | 48 | 40 | 16 | 0 | 0 | 20 | 0 | 44 | 20 | 4 | 40 | 0 | 24 | 20 | 20 | 24 |
| Rubber | BR1b | 2013 | 1 | 1 | 12 | 37 | 31 | 2 | 22 | 9 | 0 | 59 | 11 | 22 | 34 | 1 | 6 | 30 | 32 | 6 |
| Rubber | BR2b | 2013 | 0 | 0 | 21 | 18 | 18 | 2 | 3 | 23 | 0 | 21 | 16 | 1 | 27 | 0 | 0 | 26 | 17 | 1 |
| Rubber | BR3b | 2013 | 0 | 0 | 7 | 42 | 43 | 1 | 0 | 8 | 0 | 43 | 33 | 1 | 17 | 0 | 0 | 8 | 42 | 1 |
| Rubber | BR4b | 2013 | 0 | 0 | 5 | 8 | 8 | 0 | 0 | 5 | 0 | 8 | 4 | 0 | 9 | 0 | 0 | 5 | 8 | 0 |
| Rubber | HR1b | 2013 | 0 | 0 | 24 | 63 | 46 | 2 | 20 | 10 | 0 | 82 | 7 | 12 | 73 | 0 | 19 | 29 | 42 | 21 |
| Rubber | HR2b | 2013 | 0 | 0 | 7 | 28 | 25 | 6 | 0 | 8 | 0 | 30 | 2 | 20 | 16 | 0 | 5 | 8 | 23 | 7 |
| Rubber | HR3b | 2013 | 0 | 0 | 16 | 23 | 20 | 0 | 0 | 10 | 0 | 26 | 8 | 1 | 27 | 0 | 6 | 12 | 17 | 7 |
| Rubber | HR4b | 2013 | 0 | 0 | 3 | 22 | 20 | 0 | 0 | 1 | 0 | 22 | 0 | 0 | 23 | 0 | 2 | 1 | 20 | 2 |
| Rubber | BR1b | 2016 | 0 | 0 | 0 | 12 | 12 | 0 | 8 | 0 | 0 | 20 | 4 | 8 | 8 | 0 | 0 | 8 | 12 | 0 |
| Rubber | BR2b | 2016 | 0 | 0 | 48 | 52 | 24 | 0 | 0 | 20 | 0 | 52 | 16 | 0 | 56 | 0 | 28 | 20 | 24 | 28 |
| Rubber | BR3b | 2016 | 0 | 0 | 16 | 4 | 4 | 0 | 0 | 16 | 0 | 4 | 16 | 0 | 4 | 0 | 0 | 16 | 4 | 0 |
| Rubber | BR4b | 2016 | 0 | 0 | 20 | 56 | 36 | 0 | 0 | 0 | 0 | 56 | 32 | 0 | 24 | 0 | 20 | 0 | 36 | 20 |
| Rubber | HR1b | 2016 | 0 | 0 | 4 | 52 | 52 | 0 | 0 | 4 | 0 | 52 | 4 | 12 | 40 | 0 | 0 | 4 | 52 | 0 |
| Rubber | HR2b | 2016 | 0 | 0 | 48 | 84 | 48 | 0 | 0 | 12 | 0 | 84 | 16 | 44 | 36 | 0 | 36 | 12 | 48 | 36 |
| Rubber | HR3b | 2016 | 0 | 0 | 16 | 36 | 36 | 0 | 0 | 20 | 0 | 32 | 12 | 12 | 28 | 0 | 4 | 12 | 32 | 8 |
| Rubber | HR4b | 2016 | 0 | 0 | 0 | 24 | 24 | 0 | 0 | 8 | 0 | 16 | 4 | 12 | 8 | 0 | 0 | 0 | 24 | 0 |
| Rubber | HRr1b | 2016 | 0 | 0 | 24 | 20 | 16 | 0 | 0 | 4 | 0 | 36 | 4 | 24 | 12 | 0 | 12 | 4 | 16 | 20 |
| Rubber | HRr2b | 2016 | 0 | 0 | 4 | 12 | 8 | 0 | 0 | 0 | 0 | 12 | 0 | 8 | 4 | 0 | 4 | 0 | 8 | 4 |
| Rubber | HRr3b | 2016 | 0 | 0 | 16 | 40 | 48 | 0 | 0 | 16 | 0 | 48 | 12 | 24 | 28 | 0 | 4 | 12 | 36 | 16 |
| Rubber | HRr4b | 2016 | 0 | 0 | 12 | 16 | 28 | 0 | 12 | 16 | 0 | 36 | 4 | 28 | 20 | 0 | 0 | 20 | 16 | 16 |

Abbreviation of traits: Ea=Empodial appendage absent, Ms=Mouthpart sucking, PAOp= PAO present, Sp=Scales present, Ae=Abdoment IV elongated,

As=Abdoment spherical, Af=Abdoment fused, Fs=Furca straight, Fa=Furca absent, Fl=Furca long, Pd=Pigmenttaion diffuse, Pp=Pigmentation pattern,

Pa=Pigmentation absent, Pi=Pigmenttaion intensive, Am=Antennae modified, Ss=Small size, Ms=Medium size, Ls=Large size

**Supplementary Figure S1 Rarefaction curves of Collembola species from different system of each year**


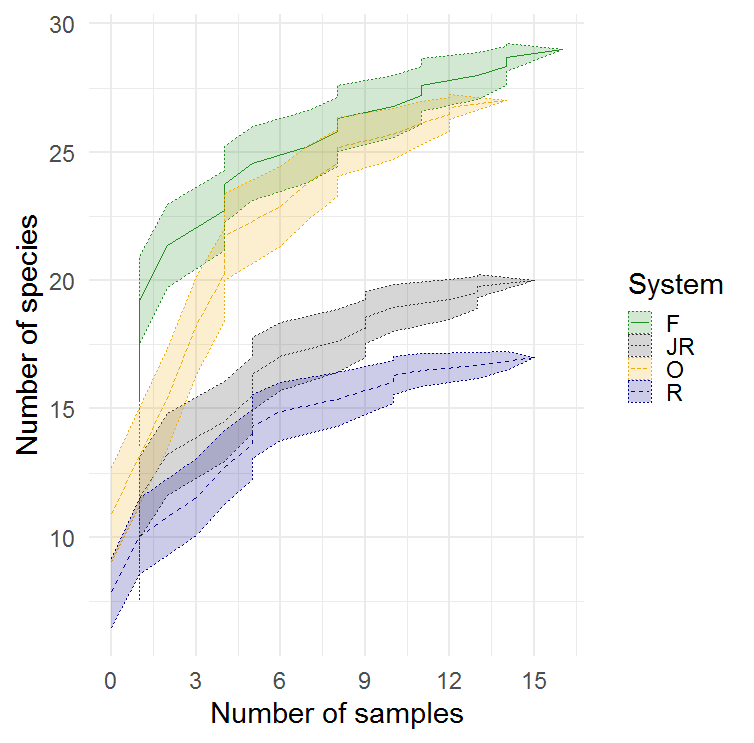

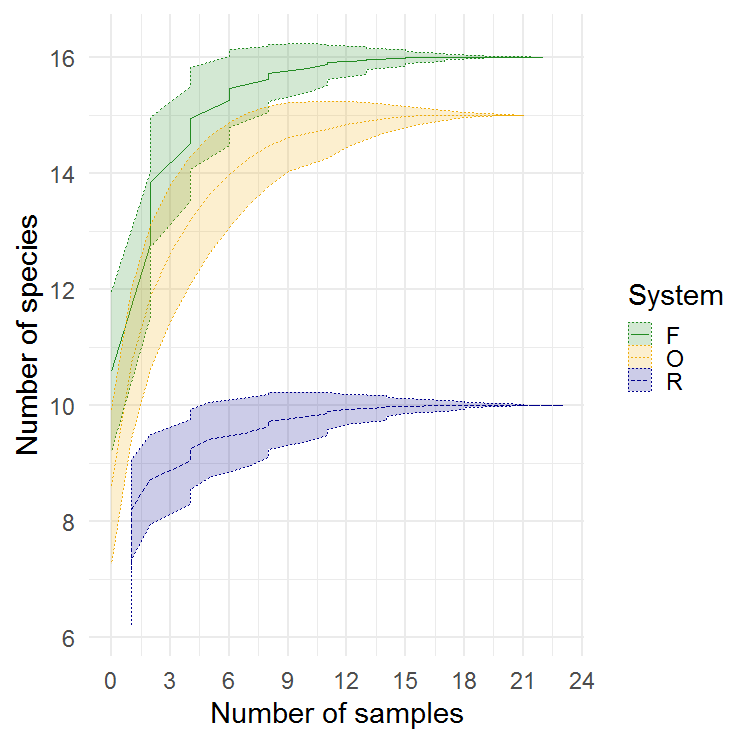


Supplementary Figure S1. Rarefaction curves of Collembola in different land-use systems in 2013 (F=Rainforest, JR=Jungle Rubber, R=Rubber, O=Oil Palm) and 2016 (F=Rainforest, R=Rubber, O=Oil Palm)

SSSupoplementary Material
